# Neural adaption in midbrain GABAergic cells contributes to high-fat diet–induced obesity

**DOI:** 10.1126/sciadv.adh2884

**Published:** 2023-11-01

**Authors:** Xiaomeng Wang, Xiaotong Wu, Hao Wu, Hanyang Xiao, Sijia Hao, Bingwei Wang, Chen Li, Katherin Bleymehl, Stefan G. Kauschke, Volker Mack, Boris Ferger, Holger Klein, Ruimao Zheng, Shumin Duan, Hao Wang

**Affiliations:** ^1^ Department of Neurosurgery of Second Affiliated Hospital and School of Brain Science and Brain Medicine, Key Laboratory for Biomedical Engineering of Education Ministry, Zhejiang University School of Medicine, Hangzhou, Zhejiang 310058, China.; ^2^ Nanhu Brain-computer Interface Institute, Hangzhou 311100, China.; ^3^ NHC and CAMS Key Laboratory of Medical Neurobiology, MOE Frontier Science Center for Brain Research and Brain Machine Integration, Key Laboratory of Precise Treatment and Clinical Translational Research of Neurological Diseases, School of Brain Science and Brain Medicine, Zhejiang University, Hangzhou, Zhejiang 310058, China.; ^4^ Institute of Hematology, Zhejiang University, Hangzhou, Zhejiang, 310058, China.; ^5^ Zhejiang Laboratory for Systems and Precision Medicine, Zhejiang University Medical Center, Hangzhou, Zhejiang 310058, China.; ^6^ Department of Anatomy, Histology and Embryology, School of Basic Medical Sciences, Health Science Center, Peking University, Beijing 100091, China.; ^7^ Department of Human Genetics and Women's Hospital, Zhejiang University School of Medicine, Hangzhou, Zhejiang 310058, China.; ^8^ Department of CardioMetabolic Diseases Research, Boehringer Ingelheim Pharma GmbH & Co. KG, Biberach, 88397, Germany.; ^9^ Global Computational Biology and Digital Sciences, Boehringer Ingelheim Pharma GmbH & Co. KG, Biberach, 88397, Germany.; ^10^ Lingang Laboratory, Shanghai 200031, China.

## Abstract

Overeating disorders largely contribute to worldwide incidences of obesity. Available treatments are limited. Here, we discovered that long-term chemogenetic activation of ventrolateral periaqueductal gray (vlPAG) GABAergic cells rescue obesity of high-fat diet–induced obesity (DIO) mice. This was associated with the recovery of enhanced mIPSCs, decreased food intake, increased energy expenditure, and inguinal white adipose tissue (iWAT) browning. In vivo calcium imaging confirmed vlPAG GABAergic suppression for DIO mice, with corresponding reduction in intrinsic excitability. Single-nucleus RNA sequencing identified transcriptional expression changes in GABAergic cell subtypes in DIO mice, highlighting *Cacna2d1* as of potential importance. Overexpressing CACNA2D1 in vlPAG GABAergic cells of DIO mice rescued enhanced mIPSCs and calcium response, reversed obesity, and therefore presented here as a potential target for obesity treatment.

## Teaser

Cacna2d1 in the vlPAG GABAergic cells is identified as a potential target for treatment of obesity.

## INTRODUCTION

Obesity is one of the most serious global public health concerns as it links to many other related diseases such as heart disease, diabetes, and stroke (*1*). Millions of people are currently affected by obesity that is often linked to excessive calorie intake. Such a factor is clearly being exacerbated by the low cost, high availability, and relentless media promotion of high-calorie foods such those rich in fat or sugar. Once it takes hold, obesity is notoriously difficult to treat. Diet and lifestyle changes are effective avenues of achieving weight loss, but the results are often temporary, with many people regaining weight within 5 years (*2*, *3*). This is possibly due to energy homeostasis, including energy intake and expenditure, being tightly controlled by the central nervous system (CNS), and that the remodeling of this system by high-fat diet (HFD) is difficult to be reversed (*4*).

The CNS, particularly the hypothalamus including the arcuate nucleus (ARC), lateral hypothalamus (LH), ventromedial hypothalamus, and paraventricular nucleus of hypothalamus (PVH), plays a key role in maintaining energy homeostasis (*5*). Prior studies in high-fat diet–induced obesity (DIO) mice have discovered that a close relationship exists between obesity and functional and structural changes in hypothalamic neural circuits. For example, HFDs have been shown to induce changes of the number of excitatory and inhibitory synaptic connections received in anorexigenic proopiomelanocortin neurons of the ARC (*6*, *7*). Similarly, the intrinsic excitability of orexigenic neurons expressing agouti-related protein in the ARC not only exhibits HFD-induced enhancement in DIO mice, but this change is also persistent and difficult to reverse, even on resumption of a normal-food diet (NFD) (*4*, *8*).

In addition to the ARC, the LH is considered as an important area that regulates energy homeostasis, particularly related to the understanding of this nucleus as a reward center (*9*). Orexin and melanin-concentrating hormone neurons are two distinct types of appetite-promoting neurons in the LH. Excitatory transmissions for both types of neurons are increased upon the feeding of a HFD (*10*–*12*). In addition to the transcriptional profile of the LH glutamatergic neurons being affected by obesity, they also exhibit greatly attenuated reward responses after HFD exposure (*13*). These prior studies suggest that HFD affects synaptic transmissions or the intrinsic excitability of hypothalamic feeding-related neurons. However, the contribution of these alterations to the pathology of obesity, as well as any changes induced by HFDs in other energy homeostasis related brain regions, remains largely unknown.

Recently, GABAergic cells in ventrolateral periaqueductal gray (vlPAG) have been identified as another key component in feeding regulation. Whole brain c-Fos protein expression surveys have revealed that vlPAG cells have been activated at different energy states (*14*, *15*). Our previous study reported that suppressing the activity of GABAergic cells in the vlPAG is sufficient to promptly induce feeding behavior in well-fed mice. Conversely, the activation of these cells interrupts food intake and consequently reduced food intake even in starved mice (*16*). Although these studies suggested a crucial role of vlPAG GABAergic cells in feeding behavior, whether the function and transcriptional profile of these neurons become altered in DIO mice, especially their roles related to energy expenditure, remains unclear.

## RESULTS

### Long-term chemogenetic activation of vlPAG GABAergic cells rescues HFD-induced obesity

**Fig. 1.**
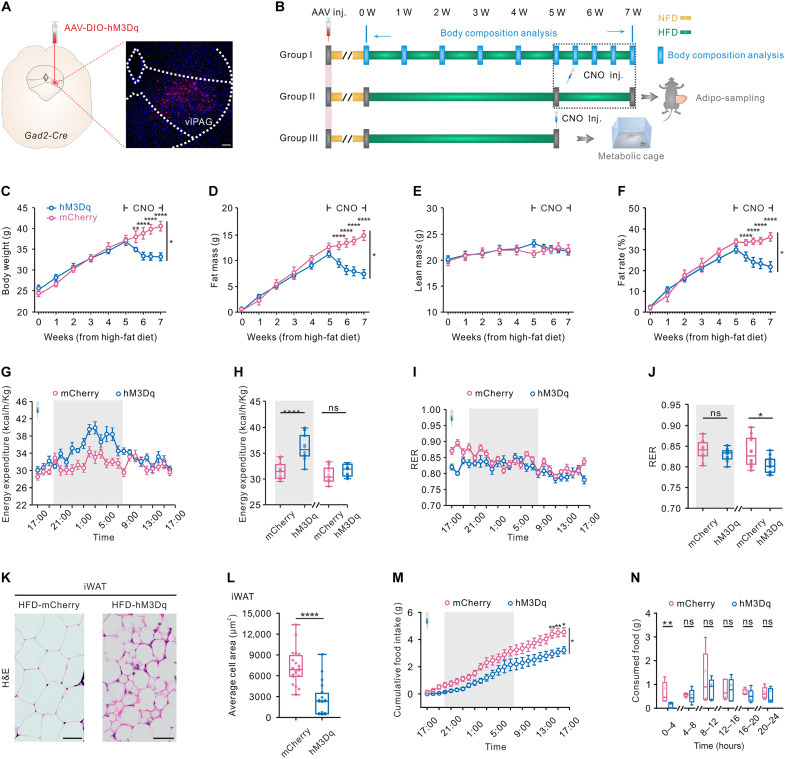
Long-term activation of vlPAG GABAergic cells reverses DIO. (**A**) Schematic and representative images. Scale bar, 100 μm. (**B**) Experimental timeline and group schematic. (**C** to **F**) hM3Dq-expression and mCherry-expression DIO mice were treated with CNO (0.25 mg/kg) intraperitoneally each day for 2 weeks. Two-way repeated-measures ANOVA was used (hM3Dq: *n* = 7 mice; mCherry: *n* = 7 mice) for body weight (*P < 0.05), fat mass (*P < 0.05), lean mass (*P* = 0.4843), and fat rate (*P < 0.05). Holm-Sidak post hoc test was used to determine time point when hM3Dq group is significantly different from control group. (**G** to **J**) hM3Dq expression and mCherry expression DIO mice were treated with CNO at 5:00 p.m. and then placed in metabolic cages for 24 hours. Energy expenditure and respiratory exchange ratios (RER) from each group of mice are shown. Bar graphs (H and J) represent averages of dark (8:00 p.m. to 8:00 a.m.) and light cycles. Shaded parts represent the dark cycle (mCherry: *n* = 12 hours averaged from seven mice; hM3Dq: *n* = 12 hours averaged from six mice; unpaired *t* test). (**K**) Representative H&E staining of the inguinal white adipose tissue (iWAT) from CNO treatment hM3Dq expression and mCherry expression DIO mice. Scale bars, 50 μm. (**L**) Average adipocyte area in iWAT in DIO mice described in (L) (*n* = 20 slides from six mice for both groups; Mann-Whitney test). (**M**) Twenty-four–hour cumulative food intake after CNO treatment for hM3Dq expression and mCherry expression DIO mice [hM3Dq: *n* = 5 mice; mCherry: *n* = 5 mice; two-way repeated-measures analysis of variance (ANOVA) followed by the Holm-Sidak post hoc test]. (**N**) Food consumption over a 4-hour period related to (M) (mCherry: *n* = 5 mice; hM3Dq: *n* = 5 mice; Mann-Whitney test). Data are represented as mean ± SEM; *P < 0.05, **P < 0.01, ****P < 0.0001. ns, not significant.

Using chemogenetics, we first wanted to examine whether the long-term activation of vlPAG GABAergic cells could reverse the overweight phenotype of DIO mice. We expressed a Cre-dependent viral construct coding for hM3Dq fused to mCherry fluorescent protein (AAV-DIO-hM3Dq-mCherry), with AAV-DIO-mCherry as control, into the vlPAG of 8-week-old *Gad2-IRES-Cre* mice to target GABAergic cells (figs. S1 and S2 and Fig. 1A). We found that 97% of *Gad2*-positive cells are also positive for *Slc32a1* by RNA scope, indicating that they are indeed GABAergic cells (fig. S1, A and B). After 1 week of recovery, the mice were switched from normal chow to HFD. Beyond that point, the body weight, fat mass, and lean mass of mice were monitored regularly using a small-animal magnetic resonance imaging (MRI) (Fig. 1, A and B). After 5 weeks on HFD, Clozapine-N-oxide (CNO) (0.25 mg/kg) was injected intraperitoneally once a day at 5:00 p.m. for a period of 2 weeks. Before CNO treatment, both groups of mice fed with a HFD showed remarkable increases in body weight, fat mass, and body fat rate but not the lean mass when compared to those of the baseline. Upon CNO treatment, the mice expressing hM3Dq began to lose body weight and fat mass quickly and continuously, while such a change was not observed in the control group (Fig. 1, C to F).

We then wanted to uncover the potential mechanism by which such a reduction of body weights had occurred during chemogenetic activation of vlPAG GABAergic cells. Body weight homeostasis is generally determined by two main factors, energy intake and energy expenditure. We tested the 24-hour level of high-fat food consumption 1 week before (baseline), 1 day after, 1 week after, and 2 weeks after CNO treatment (fig. S3A). Results showed that the amounts of high-fat food consumed were markedly lower in hM3Dq-expressing *Gad2-Cre* mice at different time points after CNO treatment as compared to the baseline levels, while the control group did not show such a reduction of food intake after CNO injection (fig. S3, B and C). We also found that 2 weeks of CNO treatment in hM3Dq-expressing DIO mice was sufficient to restore the body weight to that of age-matched mice fed with a NFD (fig. S3D).

As a next step, we explored whether activation of GABAergic neurons of vlPAG affect energy expenditure in mice. After CNO treatment at 5:00 p.m., hM3Dq-expressing and mCherry-expressing mice were tested for 24 hours in metabolic cages (Fig. 1B). Chemogenetic activation of GABAergic neurons in vlPAG resulted in a significantly increased calorie expenditure during the night but not during daytime (Fig. 1, G and H). CNO injections did also decrease the respiratory exchange rate (RER) in hM3Dq-expressing mice when compared to mCherry-expressing control mice during daytime (Fig. 1, I and J). The reduction of RER after chemoactivation of vlPAG GABAergic neurons suggested that basic energy resources had shifted from carbohydrates to fats. The reduced fat mass could be a result of adipose tissue browning, which is highly related to Uncoupling protein 1 (UCP1) signaling. Therefore, after 2 weeks of CNO treatment, we collected adipose tissues from interscapular brown adipose tissue (iBAT), inguinal white adipose tissue (iWAT), and epididymal white adipose tissue (eWAT) in hM3Dq-expressing and control mice and quantified the mRNA level of *Ucp1* by reverse transcription quantitative polymerase chain reaction (qPCR). Remarkable increase of *Ucp1* mRNA was observed in iWAT but not iBAT and eWAT groups (fig. S4, A to C). Consistently, long-term chemogenetic excitation of vlPAG GABAergic cells significantly increases the UCP1 protein level in iWAT of DIO mice (fig. S4, D and E). We performed hematoxylin and eosin (H&E) staining of iWAT collected from CNO-treated hM3Dq-expressing and mCherry-expressing DIO mice. We observed that the long-term chemogenetic activation of vlPAG GABAergic cells had induced a significant reduction of average adipose cell area and remarkable iWAT browning (Fig. 1, K and L). After CNO treatment, the reduction of 24-hour food intake in hM3Dq-expressing mice was majorly attributed by a remarkable decrease of food consumption within the first 4 hours (Fig. 1, M and N).

### vlPAG GABAergic cells display increased silencing during refeeding of high-fat food in DIO mice

**Fig. 2.**
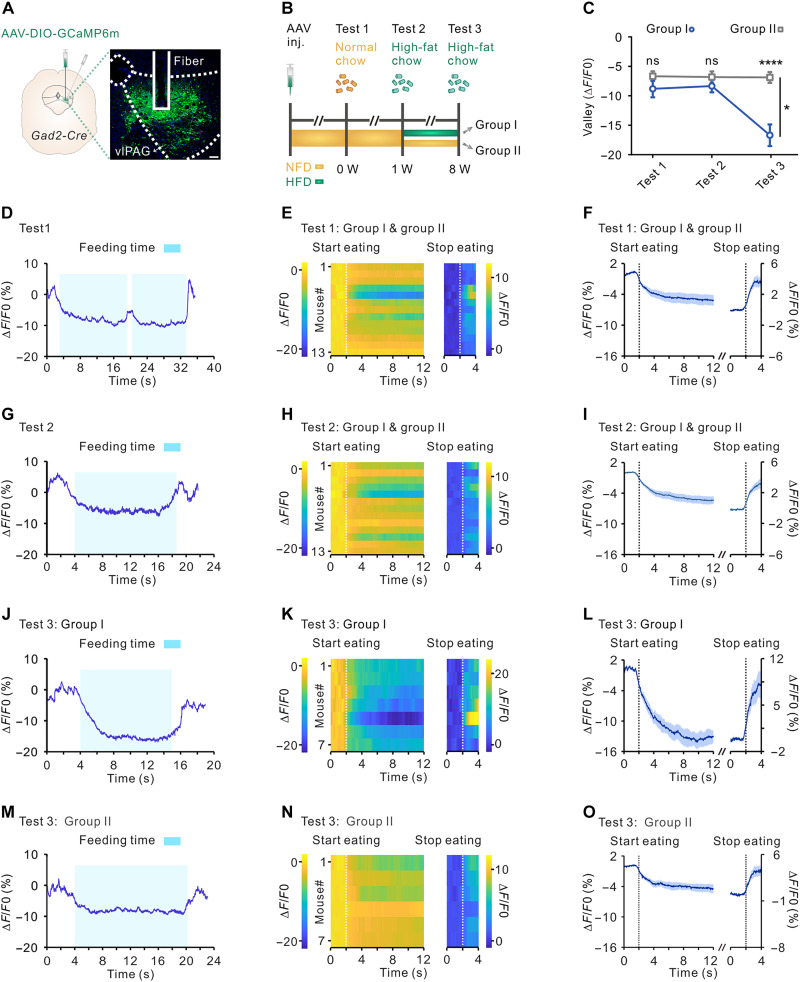
Calcium imaging of vlPAG GABAergic cells during refeeding of normal chow or high-fat chow. (**A**) Schematic for expressing GCaMP6m. Scale bar, 100 μm. (**B**) Experimental timeline and group schematic. (**C**) Amplitude of Ca^2+^ signaling in vlPAG GABAergic neurons described in (C) to (N) (test 1 group I: *n* = 7 mice, test 1 group II: *n* = 6 mice, test 2 group I: *n* = 7 mice, test 2 group II: *n* = 6 mice, test 3 group I: *n* = 7 mice, test 3 group II: *n* = 6 mice). Data are represented as mean ± SEM, and two-way repeated-measures ANOVA (*P < 0.05) followed by the Holm-Sidak post hoc test was used (*P* = 0.5746 for test 1, *P* = 0.7859 for test 2, and ****P < 0.0001 for test3). (**D** to **F**) Sample recording trace (D), heatmaps (E), and mean GCaMP6m signal (F) of all mice (group I and group II) aligned to the initiation and termination of feeding corresponding to (D) (*n* = 13 mice) in vlPAG GABAergic neurons in response to normal chow stimuli in test 1. (**G** to **I**) Sample recording trace (G), heatmaps (H), and mean GCaMP6m signal of all mice (I) showing the Ca^2+^ signals in vlPAG GABAergic neurons in response to first time high-fat chow exposure. (**J** to **L**) Sample recording trace (J), heatmaps (K), and mean GCaMP6m Ca^2+^ signal (L) of group I (*n* = 7 mice) in response to high-fat chow stimuli after being fed with HFD for 7 weeks. (**M** to **O**) Sample recording trace (M), heatmaps (N), and mean GCaMP6m Ca^2+^ signal (O) of group II (*n* = 6 mice) in response to high-fat chow stimuli after being fed with NFD for 7 weeks.

We considered whether neural adaption occurred in the vlPAG GABAergic cells in DIO mice. To answer this question, we transduced a Cre-dependent viral construct coding for the calcium-activated green fluorescent protein GCaMP6m (AAV-EF1α-DIO-GCaMP6m), into the vlPAG of *Gad2-Cre* mice unilaterally. Afterward, we positioned an optical fiber above the vlPAG for Ca^2+^ imaging (fig. S5 and Fig. 2A). Before recording, all 13 mice (groups 1 and 2) underwent 36-hour food deprivation to increase the feeding bouts upon test. To exclude the possibility of long-term fasting (36 hour) led to a notable weight loss and restoration of leptin and insulin sensitivity versus 24-hour fasting in the obese mice, we compared the body weight loss and glucose tolerance between 36-hour and 24-hour food deprivations in both DIO and control mice (fig. S6). We found that although 36 hours of food deprivation significantly reduced body weight in both groups when compared with 24 hours of food deprivation, the reduction of body weight between 36-hour and 24-hour food deprivations is comparable in DIO and control mice (fig. S6, A and B). In addition, the glucose tolerance test (GTT) results showed that the fasting blood glucose of DIO mice was higher than that of the control group, but there was no difference between the GTT of mice fasted for 24 hours and that of mice fasted for 36 hours in both the DIO and control groups (fig. S6, C and D).

Imaging sessions measuring calcium signaling were recorded in mice exposed to normal chow on week 0 and to high-fat food on week 1 and week 8 (Fig. 2, B and C). We found that in the free-feeding assay, the Ca^2+^ signaling in vlPAG GABAergic cells was markedly and persistently suppressed once the mice had commenced eating and then returned to near-baseline levels when mice completed eating, for tests on both week 0 and week 1, regardless of normal chow or high-fat food (Fig. 2, D to I). These results suggest that Ca^2+^ signaling was temperately reduced and highly correlated with feeding bouts. We then divided this cohort into two groups, one fed with HFD and the other fed with NFD as control, for 7 weeks. Mice that had undergone 7 weeks of HFD showed more robust Ca^2+^ signaling decreases during refeeding of high-fat food as compared to mice that had undergone 7 weeks of NFD (Fig. 2, J to O). To exclude the possible effects of altered locomotion after fasting on Ca^2+^ signaling, we analyzed the changes in Ca^2+^ signaling during three locomotion-related behaviors: walking, grooming, and rearing of mice that underwent 36-hour food deprivation. We found that there was no notable change in Ca^2+^ signaling during the occurrence of any of the three behaviors (fig. S6, E to M). We next found when DIO mice were refed a normal chow diet, the Ca^2+^ signals of the vlPAG also presented a notable reduction during eating bouts, and this reduction was comparable to the Ca^2+^ signal changes induced by refeeding with HFD for DIO mice (fig. S7). These results illustrate that when mice were on 7 weeks of HFD and became obese, the GABAergic neuronal activity of vlPAG is inhibited more pronounced at the onset of eating both of high-fat food and normal chow (Fig. 2C and fig. S7E). No substantial differences of number of feeding bouts, feeding bout time, and total time spent on feeding during Ca^2+^ signaling recording were observed between group 1 and 2 in test 3 (fig. S8).

### Synaptic transmission and intrinsic properties of vlPAG GABAergic cells were altered in DIO mice

**Fig. 3.**
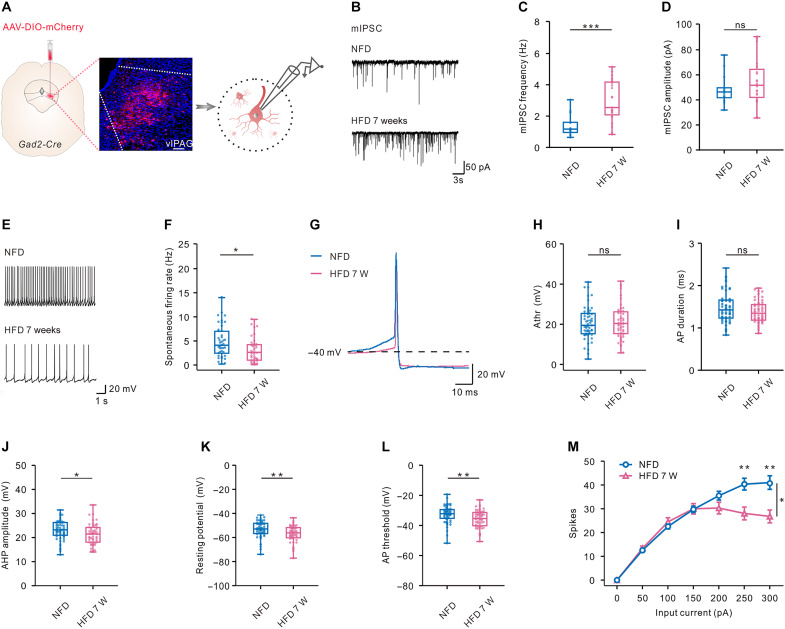
GABAergic synaptic transmission and intrinsic properties of vlPAG GABAergic cells were altered in DIO mice. (**A**) Diagram showing virus injection (left) and electrophysiological recording (right). Scale bar, 100 μm. (**B**) Representative traces of mIPSCs recorded in vlPAG GABAergic neurons from mice that had undergone 7 weeks of NFD or HFD. (**C**) Average mIPSC frequency from the two groups (NFD: *n* = 15 cells from three mice, HFD 7 W: *n* = 21 cells from three mice, ***P < 0.001, Mann-Whitney test). (**D**) Average mIPSC amplitude from the two groups (*P* = 0.247, unpaired *t* test). (**E**) Representative traces of spontaneous action potential (AP) firing. (**F**) Spontaneous AP firing rate of vlPAG GABAergic neurons from mice that had undergone 7 W NFD or HFD (NFD: *n* = 43 of 53 cells from eight mice, HFD 7 W: *n* = 43 of 52 cells from nine mice; *P < 0.05, Mann-Whitney test). (**G**) Representative traces of single AP. Dot lines represent −40 mV. (**H** to **L**) Activation threshold (H, *P* = 0.74, unpaired *t* test), AP duration (I, *P* = 0.063), AHP amplitude (J, *P < 0.05, unpaired t test), resting potential (K, **P < 0.01, Mann-Whitney test), and AP threshold (L, **P < 0.01, Mann-Whitney test) of vlPAG GABAergic neurons (NFD: *n* = 57 cells from eight mice, HFD 7 W: *n* = 52 cells from nine mice). (**M**) The number of evoked APs against injected currents (data are represented as mean ± SEM, *P < 0.05, **P < 0.01, two-way ANOVA followed by Holm-Sidak post hoc test).

The reduction of the neural activity of vlPAG GABAergic cells during refeeding in DIO mice could be a result of modified synaptic transmission or an alteration of the intrinsic excitability of these cells in DIO mice. To test these possibilities, we injected AAV-DIO-mCherry virus into the right vlPAG of adult *Gad2-Cre* mice to label GABAergic neurons. After 7 weeks of either NFD or HFD, the miniature postsynaptic inhibitory currents (mIPSCs) of GABAergic neurons in vlPAG were recorded in acute brain slices (Fig. 3A). We found that the frequency of mIPSCs was significantly increased in DIO mice as compared to NFD mice (*P* < 0.001), whereas the amplitude of mIPSCs remained unchanged (Fig. 3, B to D), suggesting that HFD may increase inhibitory synaptic transmission onto vlPAG GABAergic neurons through a presynaptic mechanism. To investigate how fast a HFD would affect synaptic function, we performed the same procedure of viral expression and electrophysiological measurements as previously described after 1 week of NFD or HFD. We found that although 1 week of HFD had not remarkably increased body weight (fig. S9A), changes of mIPSC frequency and amplitude were already in evidence (fig. S9, B to D). These results suggest that such neural adaptations precede body weight change. We also found that the spontaneous firing rate of vlPAG GABAergic cells was remarkably lower in DIO mice than in control mice (Fig. 3, E and F). The changes in spontaneous firing rates were already evidenced after 1-week HFD (fig. S9, E and F).

Besides the synaptic alterations, concurrent changes in the intrinsic excitability of postsynaptic neurons may also occur. When comparing intrinsic properties, although we observed no changes in activation thresholds and action potential (AP) duration (Fig. 3, G to I), the after-hyperpolarization (AHP) amplitude, resting membrane potential (RMP), and AP threshold were all significantly altered in DIO mice (Fig. 3, J to L). In addition, while the number of neuronal discharges in DIO mice was comparable to the control group at a current intensity input of less than 250 pA, noteworthy decreases were observed at current intensity inputs from 250 to 300 pA (Fig. 3M). All data indicated that the intrinsic excitability of GABAergic neurons in vlPAG were reduced remarkably in DIO mice. No obvious changes in intrinsic excitability were observed in mice after 1 week of HFD (fig. S9, G to M).

Given that long-term chemogenetic manipulation is capable of rescuing HFD-induced obesity, we explored whether such manipulation could also enhance the neural activity of vlPAG GABAergic cells (fig. S10). We performed c-Fos immunostaining in hM3Dq-expressing mice after single-dose or constitute 2 weeks of CNO treatments in both DIO and control groups. In DIO mice, when compared to CNO administration once, 2 weeks of CNO treatments significantly increased c-Fos expression levels in hM3Dq-expressing neurons (fig. S10, A and B). However, there was no difference in c-Fos expression levels between single CNO administration and 2 weeks of CNO administration in control mice fed with NFD (fig. S10, C and D). These results suggest that long-term chemogenetic activation increased the excitability of vlPAG GABAergic cells in obese mice.

### HFD-induced transcriptional changes in vlPAG GABAergic cells revealed *Cacna2d1* as critical player in neuronal homeostasis

**Fig. 4.**
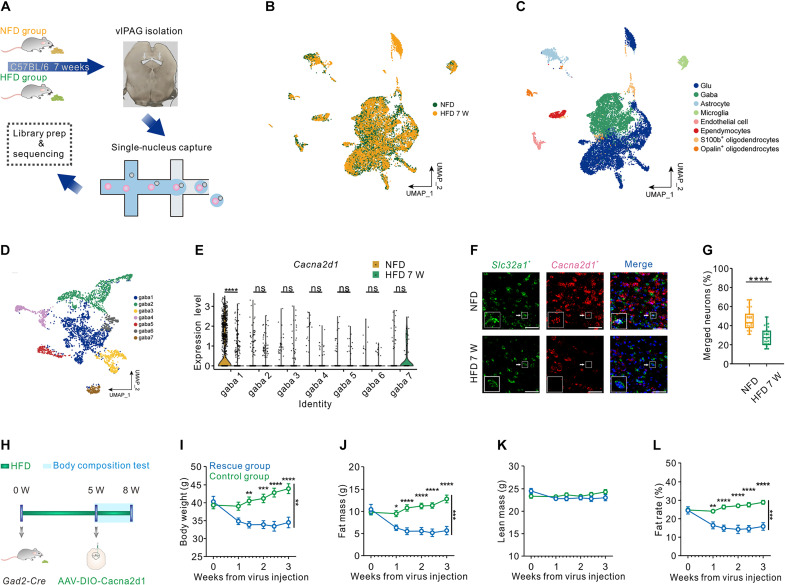
Transcriptional expression changes in vlPAG GABAergic cells in DIO mice. (**A**) Schematic representation of the workflow. (**B**) UMAP visualization of 14438 cells (7625 cells for NFD control group and 6813 cells for DIO group). (**C**) UMAP visualization of eight transcriptionally distinct clusters expressing canonical markers. Related markers see fig. S11. (**D**) Unsupervised clustering of vlPAG GABA neuronal cell types represented in a UMAP plot (*n* = 3606 cells). Different cell type clusters are color coded. Related markers see fig. S11. (**E**) Violin plot showing normalized expression of *Cacna2d1* for vlPAG^GABAergic^ neuronal cell types in NFD control and DIO groups; gaba1: ****P = 9.0 × 10^−14^ (< 0.0001), gaba2: *P* = 0.9, gaba3: *P* = 0.1938, gaba4: *P* = 0.6042, gaba5: *P* = 0.3396, gaba6: *P* = 0.8732, gaba7: *P* = 0.5349, Wilcoxon. (**F**) RNA FISH costaining of *Vgat* and *Cacna2d1* mRNA in vlPAG area. Scale bars, 50 μm. (**G**) Proportion of *Vgat*-positive neurons expressing *Cacna2d1* (NFD ctr: *n* = 3900 cells from 27 slides of four mice, DIO: *n* = 3369 cells from 27 slides of four mice; ****P < 0.0001, unpaired *t* test). (**H**) Experimental timeline and schematic for over-expressing *Cacna2d1*. (**I** to **L**) Body weight (g), fat mass (g), lean mass (g), and fat rate (%) in DIO mice after the injection of AAV-Cacna2d1 or AAV-eGFP (DIO ctr: *n* = 10 mice, DIO rescue: *n* = 9 mice; data are represented as mean ± SEM). Two-way repeated-measures ANOVA comparing *Cacna2d1* treatment rescue group and control group for body weight (**P < 0.01), fat mass (***P < 0.001), lean mass (*P* = 0.4059), and fat rate (***P < 0.001). Holm-Sidak post hoc test was used to determine time point when rescue group are significantly different from control group (*P < 0.05, **P < 0.01, ***P < 0.001, ****P < 0.0001).

To explore changes in the gene expression profile of vlPAG GABAergic cells in DIO mice, we collected the vlPAG from mice fed with either 7 weeks of HFD or normal chow and performed single-cell nucleus sequencing (Fig. 4A). In total, 6813 cells from DIO mice and 7625 cells from control mice were analyzed. To determine discrete cell classes, cells were clustered on principal components and visualized via uniform manifold approximation and projection (UMAP) for subsequent feature discovery. We identified transcriptionally distinct neuronal, glial, and stromal cell classes based on canonical marker distributions (Fig. 4, B and C, and fig. S11A). GABAergic cells were further allocated into seven subgroups (Fig. 4D and fig. S11B). We revealed many up-regulated and down-regulated genes in DIO mice (table S1). Among these genes, we were particularly interested in the *Cacna2d1* (calcium channel α2δ1 subunit) that was strongly reduced in cluster 1 (Fig. 4E), since prior studies have revealed this gene as an important regulator of synaptic plasticity (*17*). Notably, the significant difference in *Cacna2d1* expression between DIO and control mice was only observed in cluster 1, which is the most dominant subtype since it accounts for 40.99% (1478 of 3606) of all vlPAG GABAergic cells. In line with the results of single nucleus RNA sequencing (snRNA-seq), we performed RNA scope and found that the expression of the *Cacna2d1* gene was reduced in vlPAG GABAergic cells of DIO mice (Fig. 4, F and G, and fig. S12, A to C).

To further confirm the specificity of the altered *Cacna2d1* expression in cluster 1, we selected another marker gene for cluster 1, *Meis2*, and performed RNA scope for *Vgat*, *Meis2*, and *Cacna2d1* in NFD and DIO mice (fig. S12, D and E). Our results suggested that the percentage of *Meis2* and *Vgat* double-positive neurons that coexpress *Cacna2d1* was significantly lower in the DIO group compared to the NFD control group, indicating a down-regulation of *Cacna2d1* expression in cluster 1 of vlPAG GABAergic cells.

**Fig. 5.**
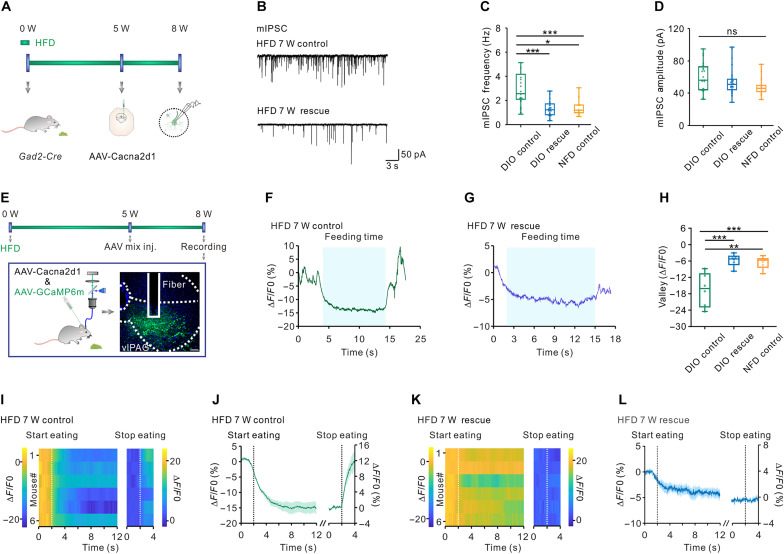
Overexpression *Cacna2d1* rescued synaptic transmission and neural activity of vlPAG GABAergic neurons in DIO mice. (**A**) Experimental timeline and schematic. (**B**) Representative traces of mIPSCs recorded in vlPAG^GABAergic^ neurons from DIO mice with CACNA2D1 overexpression or eGFP-expression. (**C**) Average mIPSC frequency from the three groups (NFD: 15 cells from three mice, DIO ctr: 30 cells from three mice, DIO rescue: 21 cells from three mice; *P < 0.05, ***P < 0.001, Kruskal-Wallis with Dunn’s multiple test). (**D**) Average mIPSC amplitude from three groups (NFD: 15 cells from three mice, DIO ctr: 30 cells from three mice, DIO rescue: 21 cells from three mice; *P* = 0.088, one-way ANOVA). (**E**) Experimental timeline and schematic for over-expressing Cacna2d1 and GCaMP6m. Scale bar, 100 μm. (**F** and **G**) Sample recording traces showing calcium signaling in response to high-fat chow stimuli in control group (F) and rescue group (G) that underwent 7 weeks HFD. Light-blue shaded parts represent feeding event. (**H**) Amplitude of Ca^2+^ signaling in different groups (DIO rescue group: *n* = 6 mice, DIO control group: *n* = 6 mice, NFD control group: *n* = 6 mice; data for NFD control group was the same from Fig. 2O. ***P < 0.001, **P < 0.01, one-way ANOVA with Tukey’s multiple test). (**I** and **J**) Heatmaps showing the Ca^2+^ signals of HFD control mice in response to high-fat chow stimuli and corresponding mean GCaMP6m signal of all mice aligned to the initiation and termination (*n* = 6 mice). (**K** and **L**) Heatmaps showing the Ca^2+^ signals of HFD rescue mice in response to high-fat chow stimuli and corresponding mean GCaMP6m signal of all mice aligned to the initiation and termination (*n* = 6 mice).

Next, we designed and unilaterally injected AAV-hSyn-DIO-Cacna2d1-pA and AAV-hSyn-DIO-eGFP (1:1) into the right-lateral region of the vlPAG of DIO *Gad2-Cre* mice to overexpress CACNA2D1 in HFD-induced obesity mice. To determine how fast AAV-Cacna2d1 viral infection can increase the expression level of CACNA2D1 in DIO mice, we explored the mRNA levels of *Cacna2d1* 1 day and 3 days after bilaterally injecting AAV-DIO-Cacna2d1 into the vlPAG brain region of DIO *Gad2-Cre* mice by RNA scope. One day after virus injection, there was already a notable increase in the proportion of *Cacna2d1* expression in *Vgat*-positive neurons. Three days after virus injection, the expression of *Cacna2d1* was further significantly increased when compared to 1 day after virus injection (fig. S13, A and B). To test how long after overexpression of *Cacna2d1* could affect food intake in DIO mice, we targeted AAV-hSyn-DIO-Cacna2d1-pA and AAV-hSyn-DIO-eGFP (1:1) and AAV-hSyn-DIO-eGFP as control into the right-lateral region of the vlPAG of DIO *GAD2-Cre* mice. After 12 hours of recovery, we tested 24-hour food consumption during three consecutive days. The results showed that although there was no change in food consumption on day 1 in the CACNA2D1 overexpression group, the amount of high-fat food consumed was significantly reduced on day 2 and day 3 after virus injection. Such a reduction in food intake could not be observed in the control group (fig. S13, C and D). One week after viral transduction, we observed a remarkable reduction of body weight, adipose tissue, and fat level, but not lean mass, of DIO mice after CACNA2D1 overexpression (Fig. 4, H to L). The reduction of body weight in CACNA2D1 overexpression DIO mice was related to a decrease of food intake and increased iWAT browning (fig. S13, E to H). After 3 weeks of CACNA2D1 overexpression, DIO mice showed a decrease in body weight to comparable levels of age-matched mice that had been fed with a NFD (fig. S14A). The reduced body weight in CACNA2D1 overexpression DIO mice was associated with a reduction of the mIPSC frequency but not mIPSC amplitude in vlPAG GABAergic cells (Fig. 5, A to D). In contrast, CACNA2D1 overexpression in mice with NFD did not change their body weights (fig. S14B).

We next asked whether and how fast overexpression of CACNA2D1 could rescue the intrinsic excitability changes of GABAergic neurons in vlPAG of DIO mice. Because of the fluorescence induced by virus expression being barely viewable 3 days after virus injection, we recorded the electrophysiological properties of vlPAG GABAergic cells 5 days after CACNA2D1 overexpression (fig. S15A). At this time point, we found that the frequency of mIPSCs was significantly reduced, while the amplitude remained unchanged when compared with those in DIO control mice (fig. S15, B to D). Consistently, the spontaneous firing rate was remarkably higher in CACNA2D1-overexpressed DIO mice (fig. S15, E and F). Among other intrinsic properties, we observed no changes in the AHP amplitude and AP threshold, while the activation threshold, RMP, AP duration, and *I*-*V* curve were markedly altered after 5 days of CACNA2D1 overexpression in DIO mice (fig. S15, G to M). All these data indicated that overexpression of CACNA2D1 for 5 days is sufficient to cause notable changes in synaptic transmission and intrinsic excitability.

To further explore the functional change of vlPAG GABAgeric cells under physiological condition after CACNA2D1 overexpression, we targeted AAV-hSyn-DIO-Cacna2d1-pA and AAV2/9-hSyn-DIO-GCaMP6m-pA (1:1) into the right-lateral region of the vlPAG of DIO *Gad2-Cre* mice and positioned an optical fiber above the vlPAG for Ca^2+^ imaging. After 3 weeks recovery, mice were first underwent 36-hour food deprivation and then were introduced to high-fat food (Fig. 5E and fig. S5). We found that although the Ca^2+^ signaling in vlPAG GABAergic cells of control (GCaMP6m-expressing only) DIO and CACNA2D1 overexpression DIO mice was markedly and persistently suppressed once the mice had commenced eating, CACNA2D1 overexpression remarkably restored the more robust Ca^2+^ signaling decreases in DIO mice (Fig. 5, F to L). We also found that a significant reduction of total time spent on feeding but not the number of feeding bouts or feeding bout time during Ca^2+^ signaling recording in CACNA2D1 overexpression DIO mice, when compared those in control DIO mice (fig. S14, C to E).

## DISCUSSION

In mammals, precise homeostatic control of body weight is critical for survival. This is largely achieved via a balance between energy intake and expenditure. Conversely, the unbalancing of energy homeostasis is one of the main causes of obesity and related diseases. Although previous studies have made substantial progress in delineating the brain circuits involved with energy intake, the neural regulation of energy expenditure and the specific neural remodeling that occurs after a HFD have only begun to be described more recently. Consistent with our previous results that suggested that the modulation of neural activity from vlPAG GABAergic cells could bidirectionally regulate feeding (*16*), the current study has shown that long-term chemogenetic activation of these cells was capable to rescue obesity in HFD-fed mice. While a large number of studies have highlighted the essential role of vlPAG in feeding behavior (particularly goal-directed behaviors like predation and other consummatory behaviors) (*16*, *18*–*21*), it remained largely unknown whether they had corresponding roles in energy expenditure. Here, excitation of the vlPAG GABAergic cells could not only reduce high-fat food consumption of DIO mice but also increase energy expenditure and induce iWAT browning. Notably, the evidences of enhanced UCP1 expression and adipose browning were absent in eWAT and iBAT. A previous study has suggested that eWAT browning is regulated by the PVH (*22*). The projection from vlPAG to PVH is relatively strong, but it is restricted to a specific subregion of the PVH (fig. S16). It is possible that vlPAG GABAergic cells do not directly project to eWAT browning promoting cells in the PVH, and therefore, stimulation of vlPAG GABAergic cells failed to induce browning and UCP1 expression in eWAT. Several lines of evidence suggest that the vlPAG may be involved in iBAT thermogenesis. First, the vlPAG receives extensive input from the medial preoptic area (mPOA) (*23*), a key brain area that regulates adipose thermogenesis (*24*), and monosynaptically projects to the raphe pallidus (RPa), which is a major site of sympathetic outflow to the periphery. Second, the vlPAG is activated by ambient warmth and cold (*25*, *26*). However, our tracing experiment (fig. S16) suggested that vlPAG GABAergic cells sent relatively weak inputs to the RPa. It is possible that excitation of this weak projection was not sufficient to induce iBAT browning and enhance UCP1 expression levels. Alternatively, a role of other cell types in the vlPAG, such as glutamatergic neurons, in regulating iBAT browning cannot be excluded.

One caveat in this study is what the downstream targets of vlPAG GABAergic cells for regulating feeding behavior and energy expenditure are. We found that vlPAG GABAergic neurons directly project to a number of feeding regulatory centers, including the nucleus accumbens, zona incerta, bed nucleus of the stria terminalis, LH, central amygdaloid nucleus, dorsomedial hypothalamic nucleus (DMH), PVH, paraventricular thalamic nucleus, ventral tegmental nucleus, and lateral parabrachial nucleus, as well as adipose metabolism regulatory centers, such as the DMH, mPOA, PVH, and RPa (fig. S16). The tracing results provided the anatomic basis for the role of vlPAG in controlling feeding and iWAT browning (*27*–*30*). Nevertheless, further studies are necessary to comprehensively determine downstream targets of vlPAG GABAergic cells in regulation of iWAT browning and feeding. It is important to note that local GABAergic cells might be involved in feeding and energy expenditure modulation through a local microcircuit.

Some other interesting studies have also observed that the dorsal raphe nucleus (DRN), a neighboring nucleus from the vlPAG, participates in energy homeostasis by using another mouse line, *Vgat-Cre* (*15*, *25*). Inhibition of *Vgat*-expressing GABAergic DRN not only reduces food intake but also increases the thermogenesis of iBAT (*25*), whereas excitation of these cells increases food consumption. Consistently, a recent study found a rising Ca^2+^ signal upon food acquisition in DRN *Gad2*-positive GABAergic neurons (*31*). DRN GABAergic cells are activated more significantly in DIO mice than in control mice, regardless of whether they consume normal chow or high-fat food (fig. S17). By using RNA scope, we revealed that although there may be small but obvious non-overlapping GABAergic populations, the majority of vlPAG and DRN neurons coexpress *Vgat* and *Gad2* (fig. S1, C to F). In contrast to DRN, Ca^2+^ signal is suppressed during feeding in *Gad2*-expressing vlPAG GABAergic cells and inhibition of these cells evokes feeding behavior. These results strongly suggest that GABAergic cells in the DRN and vlPAG are two functionally distinct populations that play opposite roles in feeding regulation, despite their close anatomical location. They may interact with each other to form midbrain hubs through recurrent (i.e., local GABA-GABA) connections to modulate feeding behavior since these two nuclei send reciprocal GABAergic projections to each other.

Our studies suggest that neural adaptions occur in the vlPAG in DIO mice. Several lines of evidence support this view. First, in vivo calcium signaling suggested that a HFD induced remarkable suppression of vlPAG GABAergic cells during re-feeding. Second, we observed increased mIPSC frequency on vlPAG GABAergic cells in DIO mice when compared to the control group. This form of plasticity was mediated by an increase in the probability of GABA release or in the number of functional synapses. Although the exact mechanism by which HFD-induced synaptic remodeling in the vlPAG remains unclear, metabolic and hormonal changes associated with HFD may be involved. A number of research papers have revealed that leptin, insulin, endocannabinoids, and brain-derived neurotrophic factors have been shown to mediate HFD-induced synaptic remodeling in various brain regions (*6*, *32*, *33*). The augmentation of the mIPSCs could be restored to control levels via manipulation of the activity or gene expression in vlPAG GABAergic cells. In particular, we found that overexpression of *Cacna2d1*, one gene that encodes for the voltage-dependent calcium channel auxiliary subunit α2δ-1, was sufficient to rescue HFD-induced obesity and to restore the mIPSC frequency, as well as abnormal strengthened calcium signaling during eating on vlPAG GABAergic cells. Other studies have suggested that α2δ-1 functions to facilitate the trafficking of high voltage-gated calcium channels to the cell surface and thereby increase calcium current density. Such a function is critical for GABAergic synaptic plasticity (*34*–*36*). Future studies are needed to elucidate the underlying mechanisms of how α2δ-1 modulates neural adaption within GABAergic synapses. Although we verified *Cacna2d1* as a key player in HFD-induced changes in the vlPAG, it is important to note that the sequencing depth by using snRNA-seq is one of the limitations of the current study. Other genes that were expressed differently in DIO mice need to be explored and verified through single-cell sequencing.

Together, our studies revealed an important role of vlPAG GABAergic cells in the regulation of energy homeostasis. We also highlighted that neural adaptions in the vlPAG may contribute to the pathology of obesity and identified *Canca2d1* as a potential therapeutic target for the treatment of obesity.

## MATERIALS AND METHODS

### Mice

All animal experiments were performed according to the Guidelines for the Care and Use of Laboratory Animals of Zhejiang University, and the protocol (AIRB-2021-528) was approved by the Zhejiang University animal experimentation committee. Adult male *Gad2-IRES-Cre* (JAX #019022) and C57BL/6 mice (JAX #000664) at the age of around 2 months were used. *Gad2-IRES-Cre* mice were obtained from The Jackson Laboratory. Mice were housed in a 12-hour light/dark cycle at (22 ± 1)°C and 55 ± 5% humidity and given food and water ad libitum. For HFD feeding, mice were subjected to a 60% HFD (D12492, ResearchDiets) starting from 8 weeks of age.

### Virus and stereotaxic injections

AAV-hSyn-DIO-hM3D(Gq)-mCherry-WPRE-Pa (AAV2/9, 2.04 × 10^12^ genomic copies/ml) and AAV-EF1α-DIO-mCherry (AAV2/9, 6.3 × 10^12^ genomic copies/ml) were produced by Sunbio. AAV-hSyn-DIO-eGFP-WPRE-pA (AAV2/9, 1.1 × 10^13^ genomic copies/ml), AAV2/9-hSyn-DIO-hM3D(Gq)-eGFP-WPRE-pA (AAV2/9, 1.1 × 10^13^ genomic copies/ml), AAV2/9-hSyn-DIO-GCaMP6m-WPRE-pA (AAV2/9, 2.0 × 10^12^ genomic copies/ml), and AAV-hSyn-DIO-3Xflag-Cacna2d1-pA (AAV2/9, 1.1 × 10^13^ genomic copies/ml) were produced by Haitool. AAV-hSyn-DIO-mGFP-2A-Synaptophysin-mRuby-WPRE-pA (AAV2/9, 5 × 10^12^ genomic copies/ml) was produced by brainVTA.

Briefly, mice were deeply anesthetized with sodium pentobarbital (1%, w/v) and placed in a stereotactic apparatus (David Kopf Instruments, Tujunga, CA). Viruses were unilaterally or bilaterally injected into the vlPAG: anteroposterior (AP): −4.36 mm, mediolateral (ML): ±0.42 mm, dorsoventral (DV): −3.30 mm from bregma). After injections, mice were treated with Lidocaine Ointment to aid recovery. For calcium signal recording, optic fibers [200-μm core, 0.37 numerical aperture (NA); Inper Inc.] were implanted in vlPAG.

For the *Cacna2d1* gene overexpression experiment, an 80-nl mixture of AAV-hSyn-DIO-Cacna2d1-pA and AAV-hSyn-DIO-eGFP (1:1) or a 120-nl mixture of AAV-hSyn-DIO-Cacna2d1-pA and AAV2/9-hSyn-DIO-GCaMP6m-pA (1:1) was infused unilaterally into the right-lateral region of the vlPAG. For the axon projection tracing experiment, a 70-nl of AAV-hSyn-DIO-mGFP-2A-Synaptophysin-mRuby-WPRE-pA was infused into the right-lateral region of the vlPAG.

### Chemogenetic manipulation

For chemogenetic experiments, CNO (Sigma-Aldrich) was dissolved in saline [5 mg in 10 μl of dimethyl sulfoxide and 190 μl of 0.9% NaCl) and then adjusted to 20 ml by addiction of 0.9% NaCl. CNO was injected intraperitoneally (0.25 mg per kg of body weight).

For the experiments of Fig. 1 and figs. S3, S4, and S10, AAV-DIO-hM3Dq or AAV-DIO-mCherry was unilaterally injected into the right-lateral region of the vlPAG (70 nl), and CNO was injected via intraperitoneally. In Fig. 1 (C to F and K to L) and figs. S3, S4, and S10, after virus injection, mice had been given a minimum of 1 week to recover before these procedures. CNO was then injected daily at 5:00 p.m. for constitute 2 weeks. In Fig. 1 (G to J and M and N), after injection, mice were given a minimum of 3 weeks to allow for virus infection. CNO was then injected daily at 5:00 p.m. for constitute 2 days before the metabolic monitoring. Data were discarded when viral expression was not restricted to vlPAG.

### Feeding behavior monitoring

As previously described (*16*), all mice used for feeding behavior studies were handled for 5 days before testing to minimize any factors relating to stress. For all chemogenetic experiments, CNO was applied at 5:00 p.m., and the experiment was performed in the metabolic cages (Fig. 1, M and N) or the home cage with a food hopper (fig. S3, B and C).

### Body composition analysis

Body composition analysis was performed during different treatments using MRI device (NIUMAG, QMR06-090H) to assess total body fat and the lean mass of each mouse. The fat rate is determined as the weight of adiposity divided by the body weight.

For the experiment of Figs. 1 (C to F) and 4 (I to L), male *GAD2-Cre* mice at 8 weeks with comparable body weights were used.

### Metabolic measurements

Energy expenditures were assessed using an indirect open-circuit calorimeter Oxylet Physiocage System (LE1305 Physiocage 00; LE405 O2/CO2 Analyzer; LE400 Air Supply and Swithching; Panlab, Cornella, Spain), equipped with a laser absorption O_2_ sensor and an infrared technology CO_2_ sensor. The experiment took 2 days, the first day for acclimation and the second day for testing. Indirect calorimetry recording was performed with room air flowing through each chamber at a rate of 450 ml/min. The O_2_ and CO_2_ levels were measured during 3 min sampling periods triggered every 30 min, and data were then analyzed using the METABOLISM software (v2.2.01). Oxygen consumption (VO_2_) and carbon dioxide production (VCO_2_) were expressed in milliliter per minute per kilogram. The RER was determined by the ratio VCO_2_/VO_2_. Energy expenditure (EE) was calculated according to the formula EE (kcal/day per kg) = VO_2_ × 1.44 × [3.815 + (1.232 × respiratory quotient)]. The O_2_/CO_2_ analyzer was calibrated with purified gas standards, and the food hoppers were replenished every day at the beginning of the light phase. The mean values for RER and EE of dark cycle and light cycle were compared for each group.

### Glucose tolerance test

Mice were deprived of food but had free access to water for 24 or 36 hours before the GTT. Blood glucose levels were measured by tail-snip blood sampling preinjection and 30, 60, 90, and 120 min after intraperitoneal glucose saline solution (1 g/kg body weight, glucose concentrations were adjusted accordingly to obtain equal injection volume for each mouse) injection.

### H&E staining for adipose tissue

At the end of CNO or virus treatment, mice were deeply anesthetized with sodium pentobarbital (1%, w/v) and sacrificed, followed by perfusion with standard artificial cerebrospinal fluid (aCSF). Adipose tissues were collected and stored at −80°C for follow-up experiments.

For H&E staining, adipose tissues were postfixed in 4% paraformaldehyde for 24 hours. Adipose sections were then made at 8 μm on a microtome (Leica, HistoCoreBIOCUT). Sections were dewaxed and rehydrated through xylene and decreasing concentrations of ethanol (100 to 75%) and then stained with H&E. After staining, sections were dehydrated through increasing concentrations of ethanol (75 to 100%) and xylene. In Fig. 1L and fig. S13H, the cell area of adipose tissues in the H&E staining slices was measured manually using ImageJ software.

### qPCR with reverse transcription

Total RNA from sorted adipose tissue was isolated by using Trizol (ABclonal). The RNA was reverse-transcribed by using the PrimeScript RT Master Mix (TaKaRa). Real-time qPCR was performed by using TB Green PreMix Ex Taq (TaKaRa) on CFX-96 (Bio-Rad). The following primers were used: 5′-AGGCCAACCGTGAAAAGATG-3′ (forward) and 5′-AGAGCATAGCCCTCGTAGATGG-3′ (reverse) for β-actin; and 5′-ACTGCCACACCTCCAGTCATT-3′ (forward) and 5′-CTTTGCCTCACTCAGGATTGG-3′ (reverse) for UCP1. All reactions were repeated in triplicate. The relative amount of mRNA was normalized with a housekeeping gene β-actin.

### Total protein extraction and Western blotting

Proteins were extracted from iWAT using a radioimmunoprecipitation assay lysis buffer (Beyotime, catalog no. P0013B) containing protease inhibitor cocktail (1:100; Yeason, catalog no. 20104ES03). Following homogenization by homogenizer and lyse on ice, lysates were centrifuged at 12,000*g* for 15 min at 4°C. The concentration of each sample was calculated by the Bicin-choninic Acid method, and an equal amount of protein from each sample was added an equal amount of protein loading buffer. The protein extracts were denatured by metal bath at 100°C for 10 min. Equal amounts of protein extracts were separated by 10% SDS–polyacrylamide gel electrophoresis and were transferred to PVDF membranes. The membranes were blocked for 1 hour in 5% DifcoTM Skim Milk (BD Difco, catalog no. 232100), followed by immunoblotting with the following primary antibodies: anti–α-tubulin (mouse, 1:100; Sigma-Aldrich, catalog no. T6199) and anti-UCP1 (rabbit, 1:1000; Abcam, catalog no. ab10983) at 4°C overnight. Next day, the membrane was washed three times in Tris Buffered Saline with Tween for 3 × 5 min, followed by incubation with the following secondary antibodies: horseradish peroxidase (HRP)–goat anti mouse (1:5000; Earthox, catalog no. E030110) and HRP-goat anti rabbit (1:5000; Earthox, catalog no. E030120) for 1 hour at room temperature.

### Calcium signal recording

Male *Gad2-Cre* mice were anesthetized with sodium pentobarbital (1%, w/v) and AAV-hSyn-DIO-GCaMP6m-WPRE-pA (80 nl; Fig. 2 and fig. S17), or a mixture of AAV-hSyn-DIO-Cacna2d1-pA and AAV2/9-hSyn-DIO-GCaMP6m-pA (120 nl, 1:1; Fig. 5) was injected into the right-lateral region of the vlPAG or the DRN. After virus injection, an optical fiber (200-μm core, 0.37 NA; Inper Inc.) was placed in a ceramic ferrule and inserted into the vlPAG (AP: −4.36 mm, ML: −0.42 mm, DV: −3.22 mm from bregma) or DRN (AP: −4.2 mm, ML: - 0.8 mm ML, DV: −3 mm from bregma, 15° angle from lateral). After surgery, mice were individually housed and allowed to recover for at least 1 week. To reduce the stress of mice, five consecutive days of gentle handling was given to mice before the experiment. The mice were allowed to acclimatize in the test chamber for 10 min before calcium imaging.

For Ca^2+^ imaging in the free-feeding assay, mice were deprived of food but had free access of water for 36 hours before the test to induce hunger and provoke enough feeding bouts during recording. Throughout the imaging session, mice were free to explore the arena and, depending on the test group, to consume a normal food pellet or high-fat food pellet, respectively. Mouse behavior was recorded by using overhead and side-mounted cameras. The 470-nm laser power was set to 30 to 40 μW. The GCaMP fluorescence was detected by a fiber photometry system (Thinkerbiotech, China).

### Data analysis for calcium signals

Calcium signals were analyzed with MATLAB software. The values of fluorescence change (delta *F*/*F*0) by calculating (*F* − *F*0)/*F*0, where *F*0 was the baseline fluorescence signal averaged in a time window recorded 2 s before regions of interest (ROIs). For the heatmaps and mean GCaMP6m signal figures, ROI indicated 2 s before the onset of feeding or corresponding locomotion event and lasting 10 s (feeding behavior), 5 s (walking and grooming behavior), or 2 s (rearing behavior) or 2 s before the end of feeding event and lasting 2 s, and feeding events that were at least 10 s long were be analyzed. For the amplitude of calcium signals (Figs. 2C and 5H and figs. S7E and S17G), ROI indicated the entire feeding bout, and all feeding bouts were be analyzed. The initiation and termination of feeding events were manually scored on the basis of the recorded video by using a customized MATLAB code. Data were discarded when viral expression was not restricted to vlPAG or the optic fiber was not targeted the vlPAG.

### Electrophysiology

Male *Gad2-Cre* mice aged between 10 and 15 weeks were used for whole-cell patch-clamp recording. AAV-EF1α-DIO-mCherry or AAV-hSyn-DIO-eGFP-WPRE-pA was injected into vlPAG to express fluoresce in GABAergic neurons. Mice were deeply anesthetized with pentobarbital and transcardially perfused with ice-cold cutting solution containing 110 mM choline chloride, 2.5 mM KCl, 1.3 mM NaH_2_PO_4_, 7 mM MgCl_2_·6H_2_O, 0.5 mM CaCl_2_·2H_2_O, 25 mM NaHCO_3_, and 20 mM _D_-glucose. Brains were quickly removed, and coronal sections of 250 μm with vlPAG were obtained using a Leica VT1200S vibratome. Slices were recovered at 32°C for 30 min in standard aCSF containing 125 mM NaCl, 2.5 mM KCl, 2 mM CaCl_2_·2H_2_O, 1.3 mM NaH_2_PO_4_, 25 mM NaHCO_3_, 1.3 mM MgCl_2_·6H_2_O, and 10 mM _D_-glucose, continuously bubbled with 95% O_2_/5% CO_2_, and then incubated at room temperature until transference into a recording chamber.

vlPAG neurons expressing mCherry or eGFP were visualized using an upright microscope with a 40× water immersion objective (Olympus BX51WI). Borosilicate pipettes with a resistance of 3 to 5 megohm were made from a P97 micropipette puller. Electrophysiological recording was performed using a Multiclamp 700B amplifier and Digidata 1550A with pClamp 10.6 software (Molecular Devices). Whole-cell current clamp recording was performed to assess excitability using pipettes filled with internal solutions containing 130 mM K-gluconate, 1 mM CaCl_2_·2H_2_O, 1 mM KCl, 10 mM Hepes, 1 mM MgCl_2_·6H_2_O, 11 mM EGTA, 2 mM Mg_2_ATP, and 0.3 mM Na_4_GTP (pH 7.3, 291 mOsm). Whole-cell voltage clamp recording was made to measure mIPSCs. For mIPSCs recording, the bath solution contained blockers of sodium channel currents (1 μM tetrodotoxin) and glutamatergic synaptic transmission (10 μM DNQX), and the pipette solution contained 140 mM CsCl, 10 mM Hepes, 4 mM MgCl_2_·6H_2_O, 0.5 mM EGTA, 4 mM Na_2_ATP, 0.4 mM Na_4_GTP, and 10 mM QX-314 (pH 7.3, 297 mOsm). Recordings lasted 1 to 2 min at −70 mV holding potential for mIPSCs. Cells with a stable series resistance (Rs) of <25 megohm and RMP of <−40 mV were included for data analysis. Data were filtered at 4000 Hz and digitized at 10,000 Hz.

### Data analysis for electrophysiological features

Data of current clamp recordings were analyzed using Clampfit v10.6 software (Molecular Devices). The following electrophysiological characteristics were calculated: RMP, AP threshold, AP duration, activation threshold (Athr), AHP amplitude, and number of AP spikes elicited during current step injections. Data of voltage clamp recordings were initially processed using Clampfit. mIPSCs were then detected and analyzed using MiniAnalysis software (Synaptosoft). The threshold for mIPSCs detection was 10 pA, and automatic detection was then manually verified post hoc. Statistical analysis and data plotting were performed using GraphPad Prism v9.2.

### Single-nucleus RNA-seq

#### Animal processing and dissection of the vlPAG

C57BL/6 mice (8 weeks old at the start of diet manipulation) were fed with on either NFD (*n* = 18 mice) or HFD (*n* = 18 mice) for 7 weeks. Mice were then deeply anesthetized with pentobarbital and sacrificed, followed by transcardial perfusion with ice-cold cutting solution containing 110 mM choline chloride, 2.5 mM KCl, 1.3 mM NaH_2_PO_4_, 7 mM MgCl_2_·6H_2_O, 0.5 mM CaCl_2_·2H_2_O, 25 mM NaHCO_3_, and 20 mM _D_-glucose. Brains were quickly removed, and coronal sections of 300 μm covering the whole vlPAG were obtained using a Leica VT1200S vibratome. Then, the sections were placed under a dissecting microscope (Olympus, SZ61) and dissected with surgical instruments for retinal operation. Afterward, tissues were stored at −80°C until further processing.

#### Single nucleus isolation

Nuclei were isolated using Nuclei EZ Lysis buffer (NUC-101; Sigma-Aldrich) supplemented with protease inhibitor (5892791001; Roche) and ribonuclease inhibitor (N2615, Promega and AM2696, Life Technologies).

#### Chromium 10x Genomics library and sequencing

Single-nuclei suspensions were loaded to 10X Chromium to capture 8000 single cells using a 10X Genomics Chromium Single-Cell 3′ kit (V3) according to the manufacturer’s instructions.

The following cDNA amplification and library construction steps were performed according to the standard protocol. Libraries were sequenced on an Illumina NovaSeq 6000 sequencing system (paired-end multiplexing run, 150 bp) by LC-Bio Technology Co. Ltd. (HangZhou, China). The sequencing depth is 41,126 mean reads per cell for NFD control group and 36,562 mean reads per cell for HFD 7 W group.

### Bioinformatics analysis

#### Raw data processing

Sequencing results were demultiplexed and converted to FASTQ format using Illumina bcl2fastq software (version 5.01). Sample demultiplexing, barcode processing, and single-cell 3′gene counting was conducted using the Cell Ranger pipeline (https://support.10xgenomics.com/single-cell-gene-expression/software/pipelines/latest/what-is-cell-ranger), and snRNA-seq data were aligned to the Ensembl genome GRCm38 reference genome. The R package Seurat (version 3.1.1) was used for dimensional reduction, clustering, and analysis of snRNA-seq data. Transcriptomes with more than 500 total counts and less than 20% of counts coming from mitochondrial genes were retained for subsequent analysis.

#### snRNA-seq data analysis

For the remaining cells, gene expression matrices were normalized to the total unique molecular identifier counts per cell and were log-transformed (on a base 2 scale). Dimensionality reduction was performed with UMAP using the RunUMAP function in Seurat. Differential expression gene between each cell type and biological status was obtained using the FindMarkers function in Seurat.

#### Cluster analysis for GABAergic neurons

We selected the cells which express at least one of three markers of GABAergic neuron cells (*Slc32a1*, *Gad1*, and *Gad2*) in previously identified GABAergic neurons and reclustered the remaining neuron cells using the FindClusters function in Seurat with the resolution value set as equal to 0.1. Differential expression gene between each cluster and biological status was obtained by using the FindMarkers function in Seurat.

#### FISH and immunohistochemistry

In Fig. 4 (F and G) and figs. S1, S12, and fig. S13 (A and B), mice were deeply anesthetized with sodium pentobarbital (1%, w/v) and sacrificed, followed by transcardial perfusion with saline followed by 4% paraformaldehyde (w/v). Brains were removed and postfixed in 4% paraformaldehyde for 10 to 12 hours and then were dehydrated in 30% sucrose (w/v) for at least 48 hours at 4°C. Before cutting, brains were embedded and frozen in optimum cutting temperature compound. Brain sections were constructed at 16 μm on a cryostat. RNAscope 2.5 Assay (Advanced Cell Diagnostics) was used for all fluorescence in situ hybridization (FISH) experiments according to the manufacturer’s protocols (*37*). All RNAscope FISH probes were designed and validated by Advanced Cell Diagnostics (Probe-Mm-SLC32A1, catalog no. 319191-C2; Probe-Mm-Cacna2d1, catalog no. 417141-C1; Probe-Mm-Gad2, catalog no. 439371-C1; Probe-Mm-Meis2, catalog no. 436371-C3). The slices were mounted using FluorSave Reagent (20 ml; Merck Millipore, no. 345789) with 4′,6-diamidino-2-phenylindole (DAPI; Advanced Cell Diagnostics).

In figs. S1 (A and B) and S12 (B and C), following the FISH protocol, sections were blocked for 1 hour in 5% donkey normal serum (DNS) in phosphate-buffered saline solution supplemented with 0.1% Tween-20 (PBST) and then incubated with rabbit anti-GFP (1:1000; Abcam, no. ab6556) at 4°C overnight. The following day, the sections were rocked and washed 3 × 10 min in 0.1 M phosphate buffer and then incubated with fluorophore-conjugated secondary antibody for 1 hour at room temperature (donkey anti-rabbit Alexa Fluor 488, 1:800; Invitrogen, catalog no. A21206). Antibodies were diluted in PBST containing 5% DNS.

In fig. S10, 2 hours after CNO treatment (0.25 mg per kg of body weight, i.p.), mice were deeply anesthetized with sodium pentobarbital (1%, w/v) and then sacrificed for immunohistochemistry staining. The primary antibody was anti–c-Fos (guinea pig, 1:800; Synaptic System, catalog no. 226308), and the secondary antibody was donkey anti–guinea pig Alexa Fluor cy3 (1:1000; Jackson Immuno Reasearch , catalog no. 706-165-148).

After staining, sections were then mounted using FluorSave Reagent (20 ml; Merck Millipore, no. 345789) with DAPI (Advanced Cell Diagnostics). Last, all images were acquired with 20×, 40×, or 60× objectives using an Olympus FV3000 confocal microscope. The proportion of *Vgat*-positive neurons expressing *Cacna2d1* or *Gad2*-, *Vgat*-, and *Meis2*-positive neurons expressing *Cacna2d1*, GFP labeling neuron expressing *Cacna2d1* or *Slc32a1*, and hM3Dq-eGFP labeling neurons expression c-Fos was calculated by using ImageJ software.

### Statistical analysis

In all experiments, data acquisition and analyses were performed in a blind manner. Datasets were first determined whether fit into a normal distribution model or not. When data fitted normal distribution, one-way analysis of variance (ANOVA) followed by Tukey’s post hoc test or unpaired *t* test was to determine statistical differences. When data were not normally distributed, Kruskal-Wallis followed by Dunn’s post hoc test or Mann-Whitney test was used. Two-way repeated-measures ANOVA followed by Holm-Sidak or Tukey post hoc analysis was used to evaluate the effects of long-term manipulation (Figs. 1, C to F and M, 2C, and 4, I to L, and fig. S14B). Two-way ANOVA followed by Holm-Sidak post hoc analysis was used in Fig. 3M and figs. S9M and S11M. Data were analyzed with GraphPad Prism 6 or SPSS software. Statistical significance was *P < 0.05, **P < 0.01, ***P < 0.001, and ****P < 0.0001.
