# Supplementary material for: Neural adaption in midbrain GABAergic cells contributes to high-fat diet–induced obesity
Source: Sci Adv. 2023 Nov 1;9(44):eadh2884. doi: 10.1126/sciadv.adh2884 (PMC10619925; doi:10.1126/sciadv.adh2884)
Supplement: Supplementary file 1 — Figs. S1 to S17 Table S1 [file sciadv.adh2884_sm.pdf]

Science Advances
AAAS

Supplementary Materials for

Neural adaption in midbrain GABAergic cells contributes to high-fat diet–induced obesity

Xiaomeng Wang *et al.*

Corresponding author: Hao Wang, haowang@zju.edu.cn

Sci. Adv. **9**, eadh2884 (2023)
DOI: 10.1126/sciadv.adh2884

This PDF file includes:

Figs. S1 to S17
Table S1

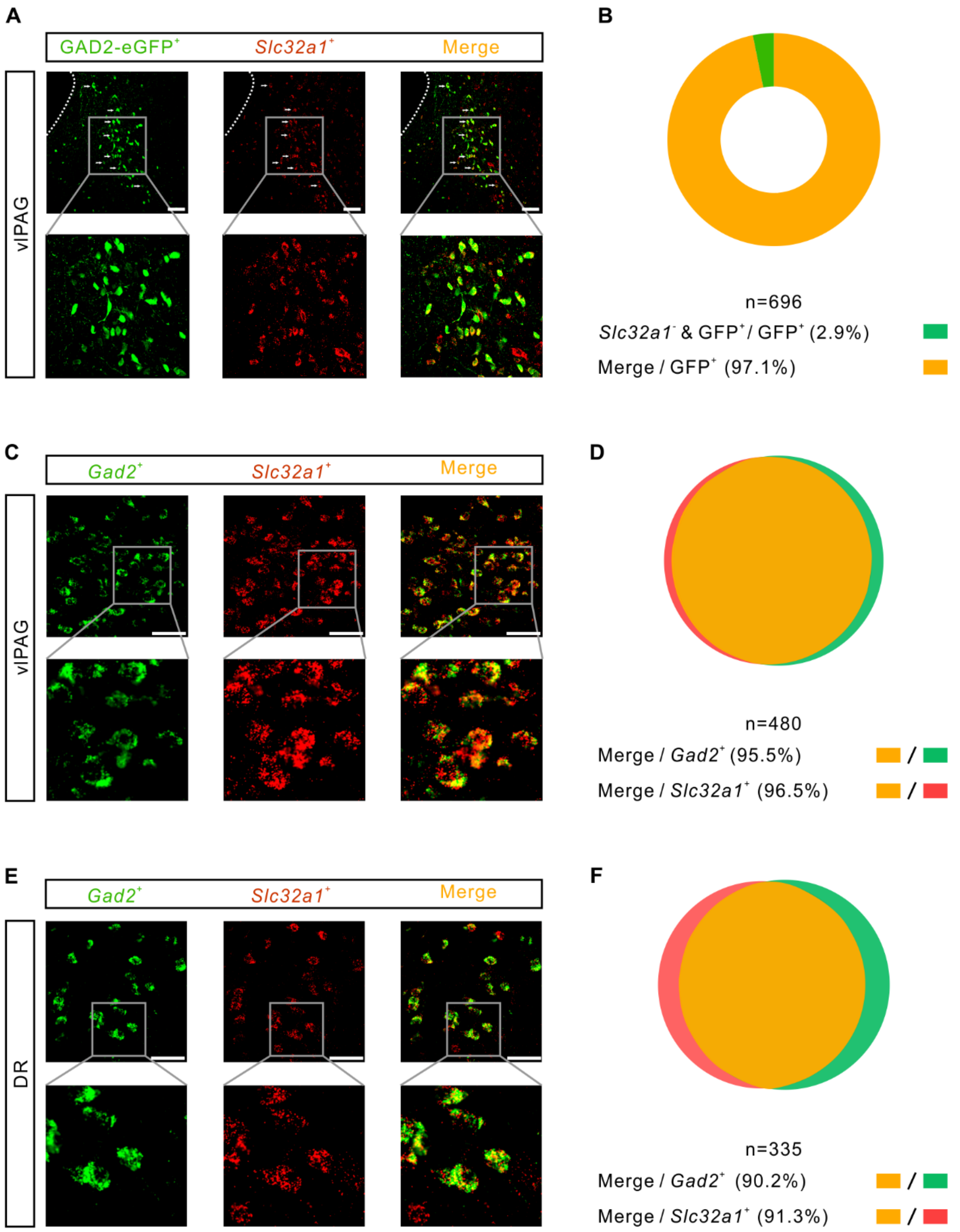

Fig. S1. ***Gad2-Cre*** **mouse line targets GABAergic neurons in the vlPAG specifically and the expressing pattern of** ***Vgat*** **and** ***Gad2*** **in the vlPAG and DRN.** **(A)** RNA FISH staining of *Slc32a1* in vlPAG area with eGFP virus expression in *Gad2-Cre* mice. Scale bars represent 100 μm. **(B)** Proportion of eGFP-positive neurons that co-expressing of *Slc32a1* (n = 696 cells from 12 slices of 4 mice).

(C) RNA FISH staining of *Slc32a1* and *Gad2* in vlPAG area. Scale bars represent 50μm. **(D)** Proportion of *Vgat*-positive neurons that co-expressing of *Gad2* or *Gad2*-positive neurons that co-expressing of *Vgat* in the vlPAG (n = 480 cells from 9 slices of 3 mice). **(E)** RNA FISH staining of *Slc32a1* and *Gad2* in DRN area. Scale bars represent 50μm. **(F)** Proportion of *Vgat*-positive neurons that co-expressing of *Gad2* or *Gad2*-positive neurons that co-expressing of *Vgat* in the DRN (n = 335 cells from 8 slices of 3 mice).

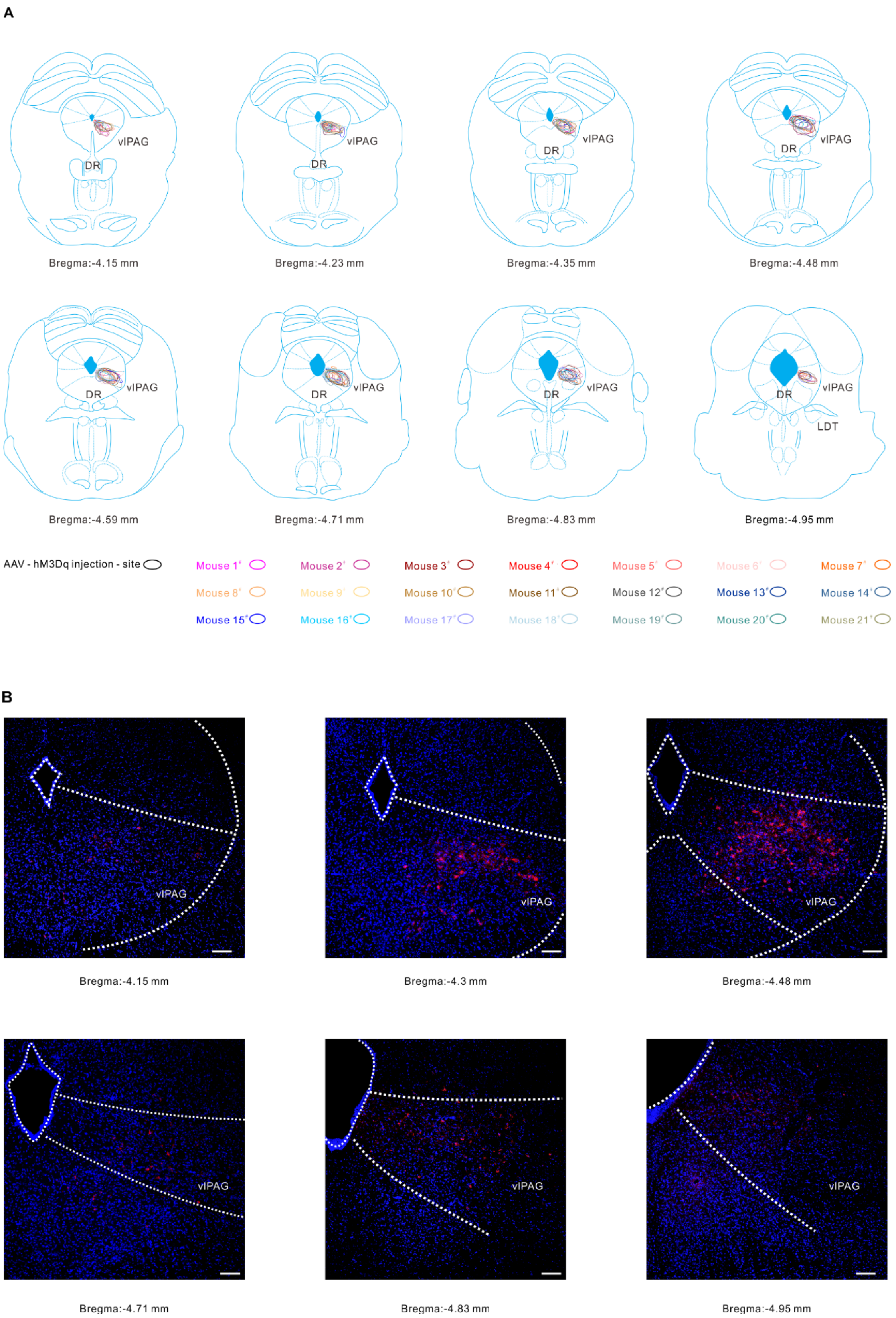

Fig. S2. Reconstructions and representative images of AAV-hM3Dq-mCherry injection sites for experiments shown in Fig. 1 and fig S3 and S4.

(A) Serial reconstructions of AAV-hM3Dq-mCherry injection sites in vlPAG (solid lines). **(B)** Serial sections showing hM3Dq-mCherry expression in the vlPAG. Scale bars represent 100 μm.

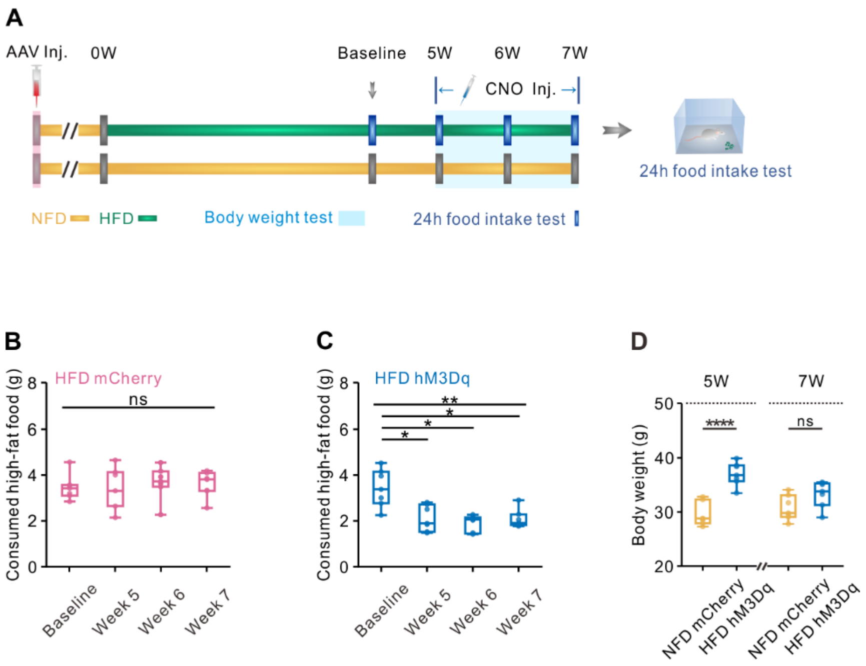

Fig. S3. Long-term chemogenetic activation of vlPAG GABAergic cells reduces food intake and reverses obesity in DIO mice.

(A) Experimental timeline and schematic. **(B-C)** 24 h food intakes before (baseline) and after CNO treatments (mCherry: n = 7 mice; hM3Dq: n = 6 mice; *p < 0.05, **p < 0.01, Kruskal-Wallis with Dunn's multiple test). **(D)** Body weights of mice fed with HFD or NFD were measured before and at the end of CNO treatment (NFD mCherry: n = 7 mice, HFD hM3Dq: n = 7 mice; ****p < 0.0001 for 5W, p = 0.067 for 7W, unpaired t test).

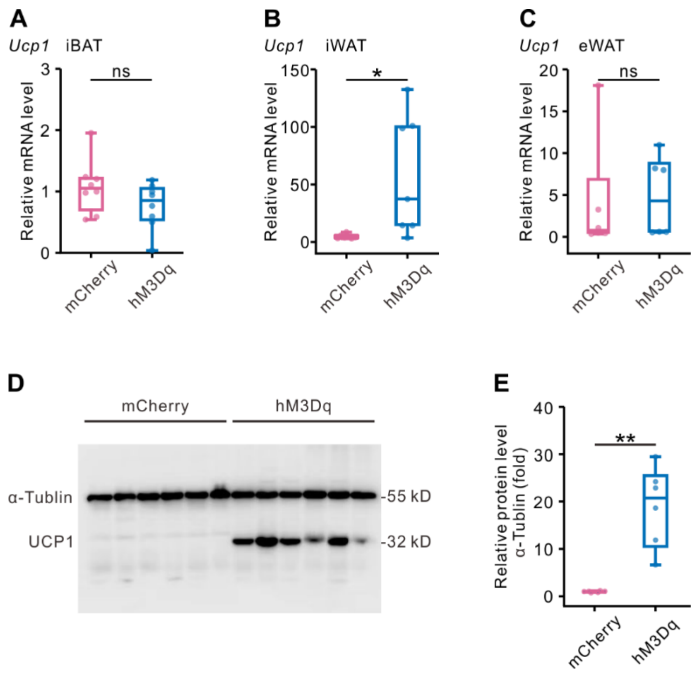

Fig. S4. Changes of UCP1 mRNA and protein level in adipose tissues after long-term chemogenetic excitation of vlPAG GABAergic cells.

(A-C) qRT-PCR profile of mRNA level of *Ucp1* in the interscapular brown white adipose (iBAT, A), inguinal white adipose tissue (iWAT, B), and epididymal white adipose tissue (eWAT, C) of the mice after 2 weeks CNO treatment (iBAT: n = 8 mice for both hM3Dq and mCherry group, iWAT: n = 7 mice for both hM3Dq and mCherry group, eWAT: n = 6 mice for both hM3Dq and mCherry group; *p < 0.05, unpaired t test or Mann-Whitney test). **(D)** Western blots of UCP1 and α-Tublin from iWAT. **(E)** The quantified ratio of UCP1/α-Tublin, n = 6 mice for both hM3Dq and mCherry group, **p < 0.01, Mann-Whitney test.

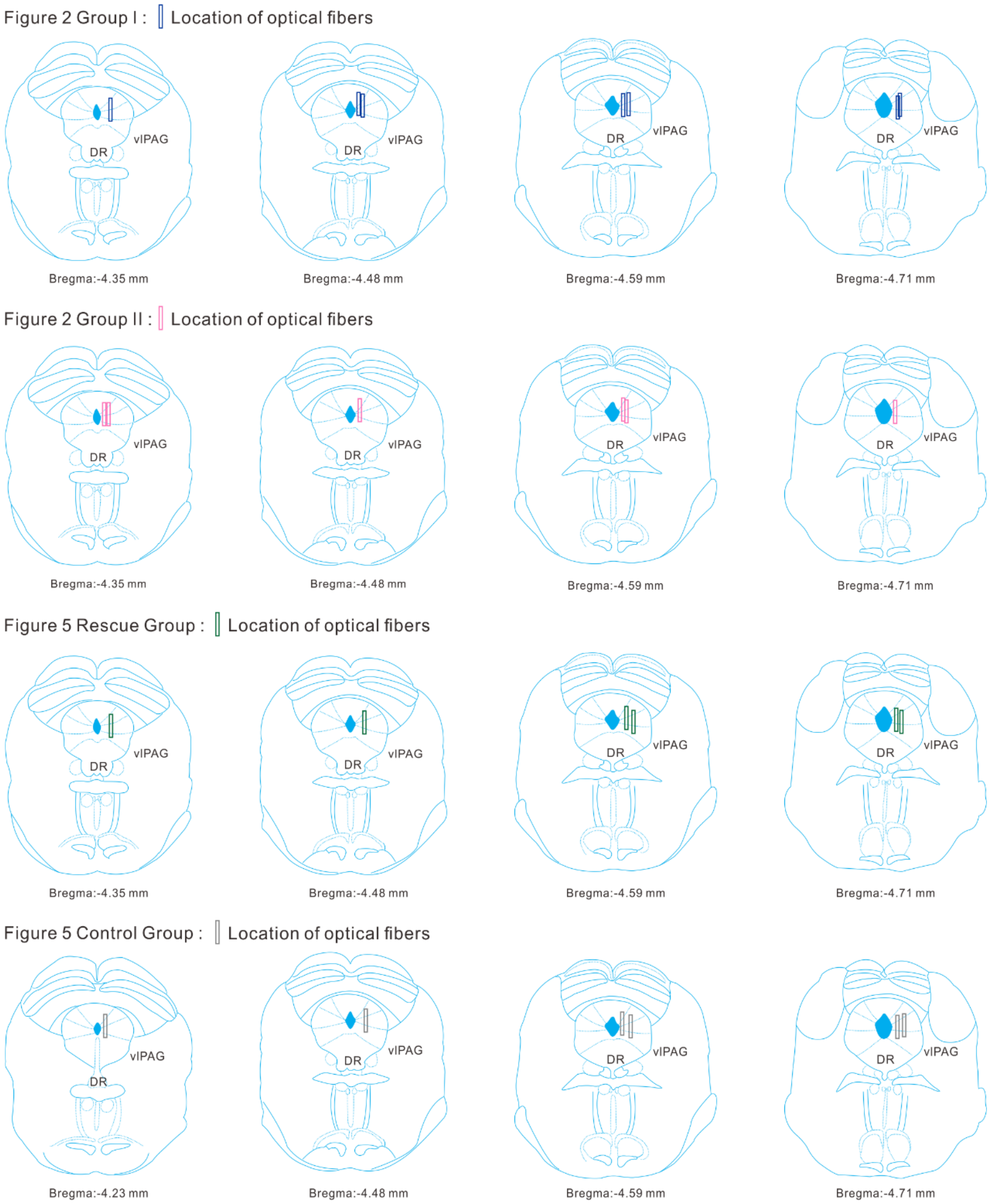

Fig. S5. Details of optical fiber locations in the vlPAG related to Figure 2, and 5.

Locations of optical fiber placement in the vlPAG for Ca^{2+} signal recording in Fig. 2 (top) and Fig.5 (bottom).

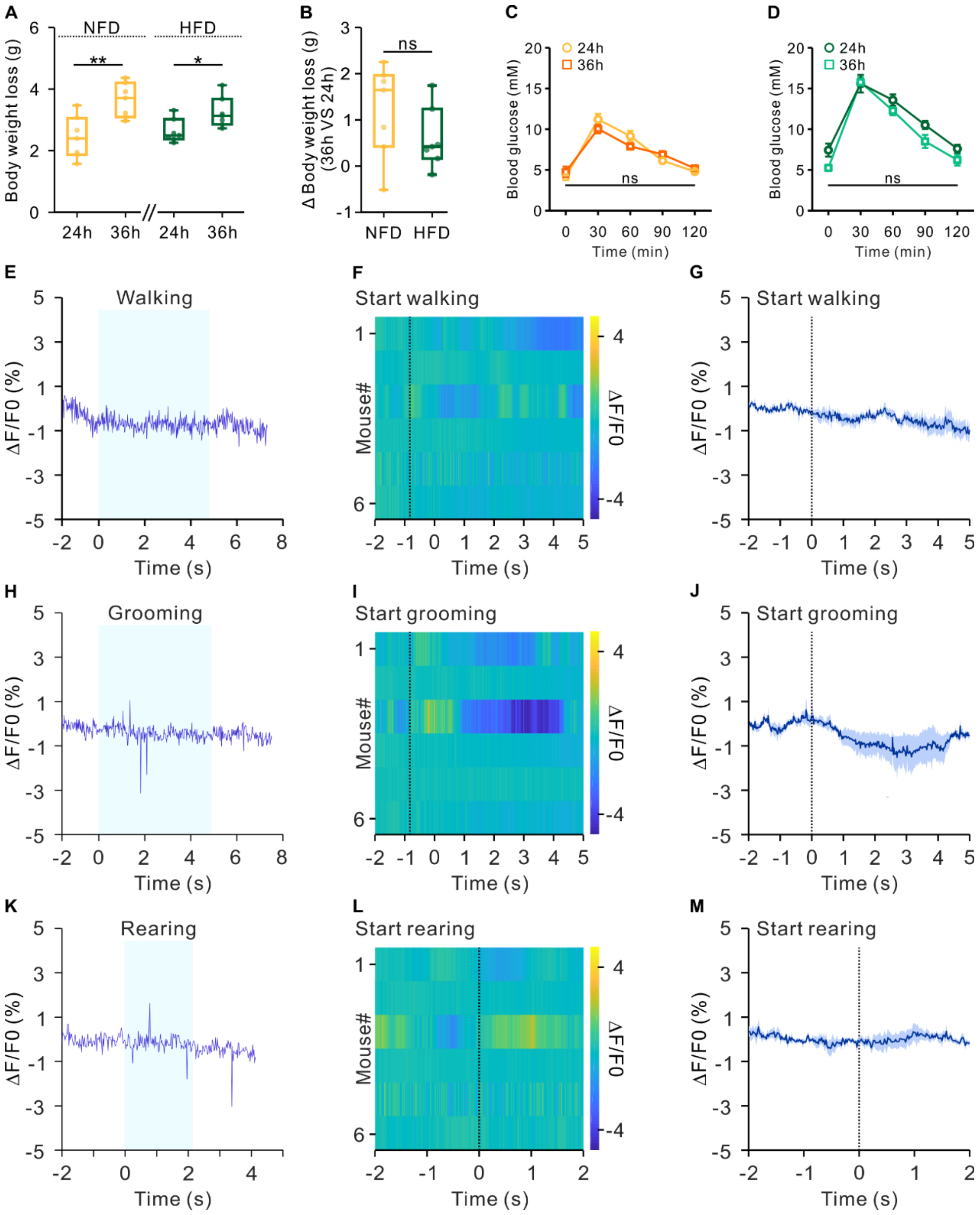

Fig. S6. Analysis of locomotion behavior during free-feeding assay of Fig. 2.

(A) Weight loss of NFD mice and DIO mice underwent 24 or 36h food deprivation; n = 7 mice for both NFD and DIO group , * $p < 0.05$, ** $p < 0.01$ unpaired t test. **(B)** Increased weight loss of NFD mice and DIO mice underwent 36h food deprivation compared with 24h; $p = 0.2047$, unpaired t test. **(C-D)**

Blood glucose levels during GTT of NFD and DIO mice underwent 24 or 36h food deprivation; n = 7 mice for both NFD and DIO group, p = 0.7742 for NFD group, p = 0.0623 for DIO group, two-way RM ANOVA. Data are represented as mean ± SEM **(E-G)** Sample recording trace (E), heat maps (F), and mean GCaMP6m signal (G) of mice aligned to the initiation of walking corresponding to F (n = 6 mice) in vlPAG GABAergic neurons. **(H-J)** Sample recording trace (H), heat maps (I), and mean GCaMP6m signal (J) of mice aligned to the initiation of grooming corresponding to I (n = 6 mice) in vlPAG GABAergic neurons. **(K-M)** Sample recording trace (K), heat maps (L), and mean GCaMP6m signal (M) of mice aligned to the initiation of rearing corresponding to L (n = 6 mice) in vlPAG GABAergic neurons.

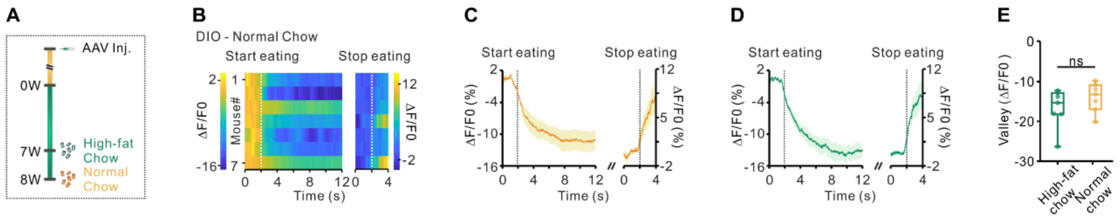

Fig.S7. Calcium imaging of vlPAG GABAergic cells of DIO mice during refeeding of normal chow or high-fat chow.

(A) Experimental timeline: The mice were fed with HFD for 7 weeks and then were tested with high-fat chow or normal chow. **(B-C)** Heat maps showing the Ca^{2+} signals in vlPAG GABAergic neurons of DIO mice in response to normal chow stimuli and corresponding mean calcium signal of all mice aligned to the initiation and termination (n = 7 mice). **(D)** Mean Ca^{2+} signal of all DIO mice aligned to the initiation and termination of feeding in response to high-fat chow stimuli. Data for high-fat chow test was same from Fig. 2L. **(E)** Amplitude of Ca^{2+} signaling in vlPAG GABAergic neurons of DIO mice in response to high-fat chow or normal chow stimuli; n = 7 mice, p = 0.2581, unpaired t test.

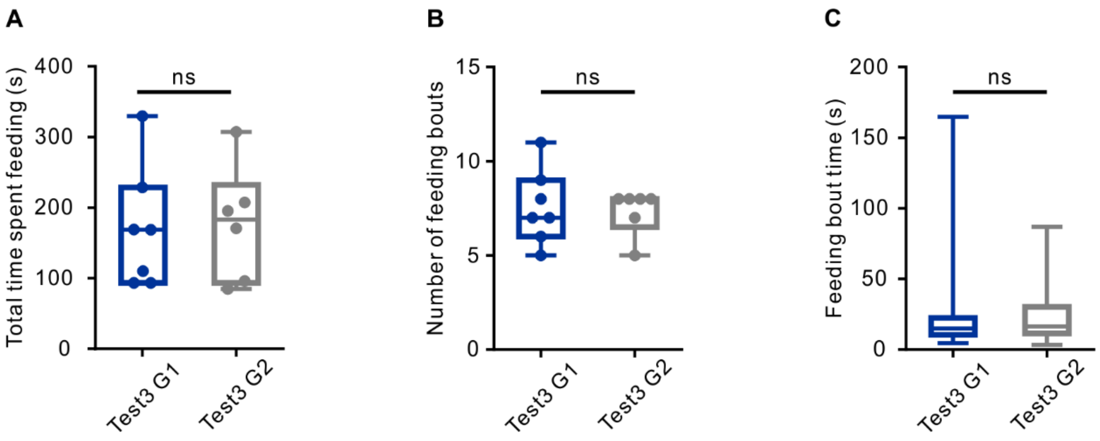

Fig. S8. Detailed analysis of feeding behavior in test3 of Fig. 2.

(A) Total time the mice spent feeding during a 10 min test (Group I: n = 7 mice, Group II: n = 6 mice, p = 0.8135, Mann-Whitney test). **(B)** Number of feeding bouts during a 10 min test (Group I: n = 7 mice, Group II: n = 6 mice, p = 0.9796, Mann-Whitney test). **(C)** The length of feeding bouts during a 10 min test (Group I: n = 52 bouts from 7 mice, Group II: n = 44 bouts from 6 mice, p = 0.3416, Mann-Whitney test). All feeding bouts were analyzed.

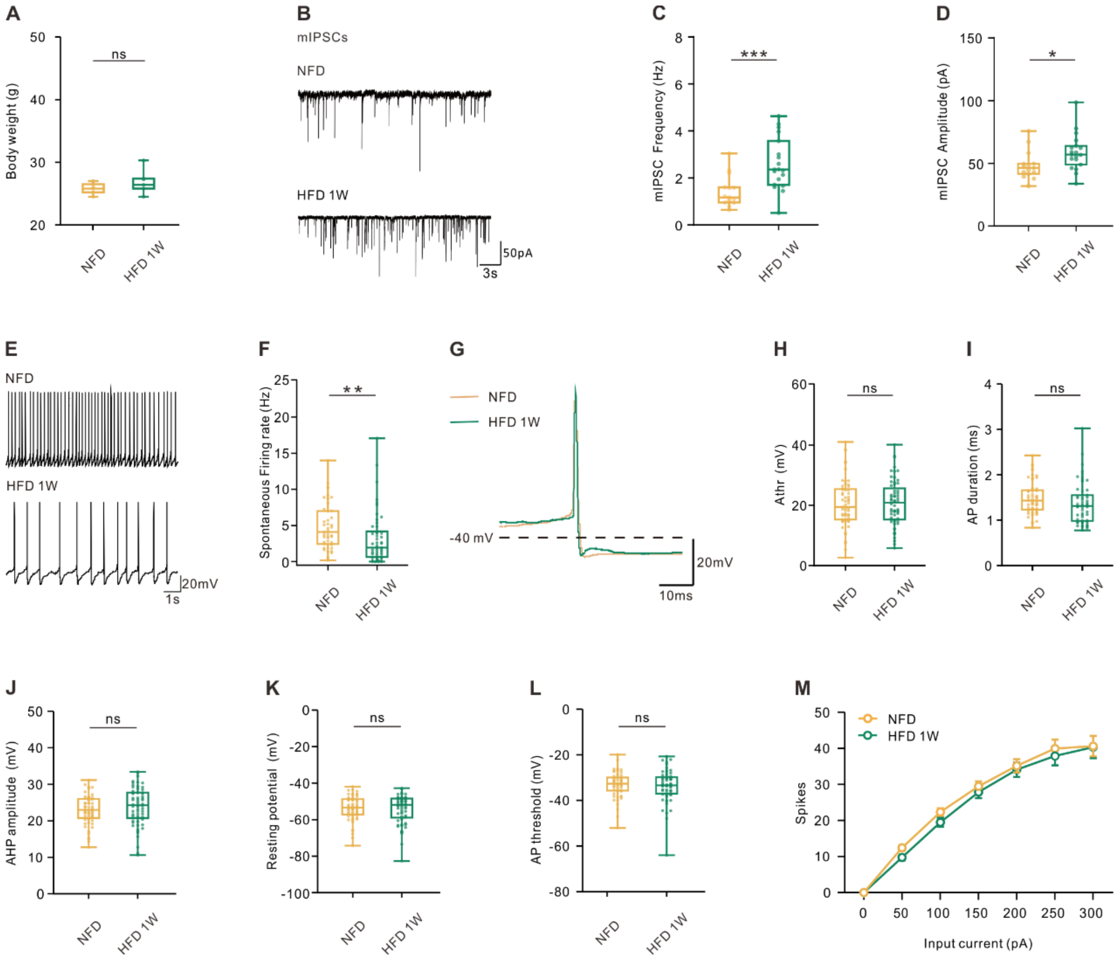

Fig. S9. Body weights and electrophysiological properties of vlPAG GABAergic cells in mice that had undergone 1W HFD.

(A) Body weights of mice fed with either 1 week of NFD or HFD (n = 7 mice for both NFD and HFD groups; p = 0.1986, unpaired t test). **(B)** Representative traces of mIPSCs recorded in vlPAG GABAergic neurons from mice that had undergone 1W NFD or HFD. **(C)** Average mIPSCs frequency from the two groups (NFD: n = 15 cells from 3 mice, HFD 1W: n = 19 cells from 3 mice, ***p < 0.001, Mann-Whitney test). **(D)** Average mIPSCs amplitude from the two groups (NFD: n = 15 cells from 3 mice, HFD 1W: n = 19 cells from 3 mice, *p < 0.05, Mann-Whitney test). **(E)** Representative traces of spontaneous action

potential (AP) firing. **(F)** Spontaneous AP firing rate of vlPAG GABAergic neurons from mice that had undergone 1W NFD or HFD (NFD: n = 43/53 cells from 8 mice, HFD 1W: n = 50/60 cells from 10 mice; **$p < 0.01$, unpaired t test). **(G)** Representative traces of single AP. Dot lines represent -40mV. **(H-L)** Activation threshold (H, $p = 0.90$, unpaired t test), action potential duration (**I**, $p = 0.09$), AHP amplitude (J, $p = 0.22$, unpaired t test), resting potential (K, $p = 0.95$, Mann-Whitney test) and AP threshold (L, $p = 0.70$, Mann-Whitney test) of vlPAG GABAergic neurons recorded from mice that had undergone 1W NFD or HFD (NFD: n = 56 cells from 8 mice, HFD 1W: n = 62 cells from 10 mice). **(M)** The number of evoked action potentials against injected currents (Data are represented as mean ± SEM, NFD: n = 56 cells from 8 mice, HFD 1W: n = 62 cells from 10 mice, $p = 0.4283$, Two way ANOVA).

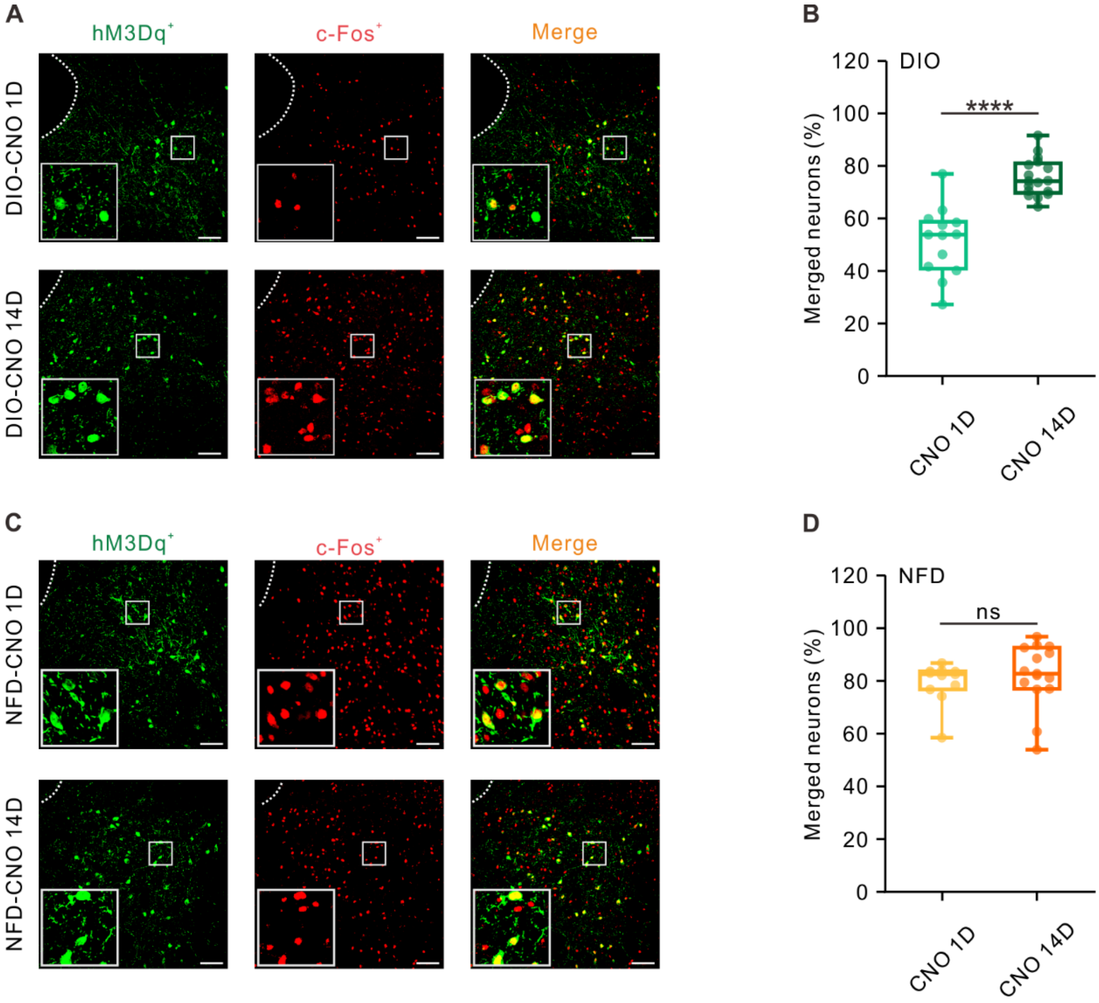

Fig. S10 C-Fos expression of vlPAG GABAergic neurons after CNO treatment. **(A-B)** Representative images and corresponding quantification of the c-Fos expression level in DIO mice after single dose or chronic (14 days) CNO treatment; CNO 1 Day: n = 620 cells from 13 slices of 4 mice, CNO 14 Day: n = 954 cells from 17 slices of 4 mice, **** $p < 0.0001$, Mann-Whitney test. Scale bars represent 100 μm. **(C-D)** Representative images and corresponding quantification of the c-Fos expression level in NFD mice after acute (1 day) or chronic (14 days) CNO treatment; CNO 1 Day: n = 462 cells from 11 slices of 4 mice, CNO 14 Day: n = 677 cells from 15 slices of 4 mice, $p = 0.4045$, Mann-Whitney test. Scale bars represent 100 μm.

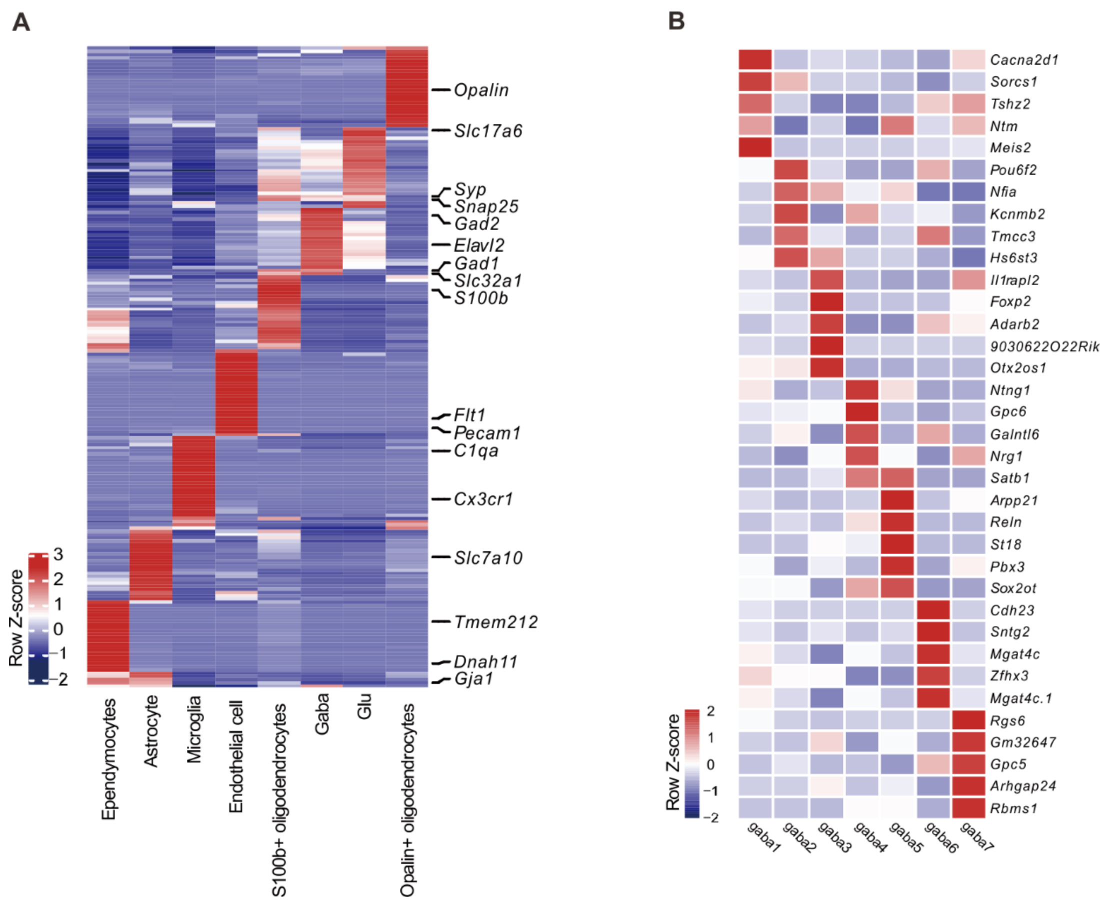

Fig. S11. Statistically defined clusters exhibit distinct expression patterns.

(A) Statistically defined clusters exhibit distinct expression patterns for all 14438 cells obtained from vlPAG. **(B)** Statistically defined clusters exhibit distinct expression patterns for all 3606 GABAergic cells.

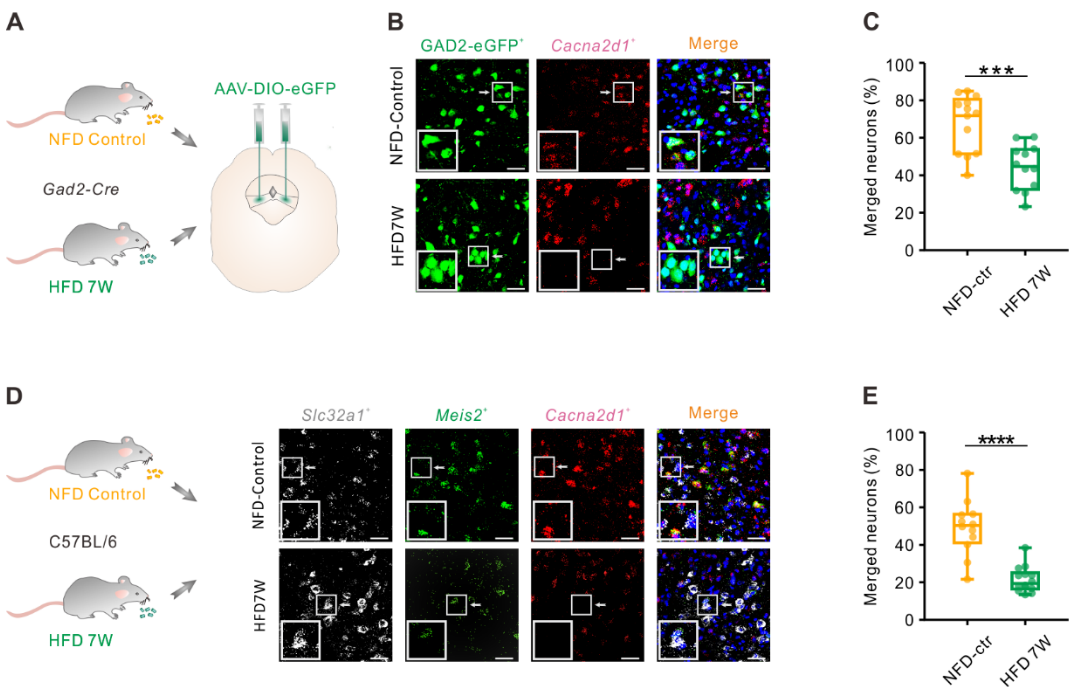

Fig.S12. Expression of *Cacn2d1* was reduced in vlPAG *Gad2*+ cells and *Vgat*+ & *Meis2*+ cells of DIO mice.

(A-B) Schematic and representative images of RNA FISH co-staining of *Gad2* and *Cacna2d1* mRNA in vlPAG area. Scale bars represent 50 μm. **(C)** Proportion of GAD2-eGFP positive neurons expressing *Cacna2d1* (NFD ctr: n = 830 cells from 13 slices of 4 mice, DIO: n = 815 cells from 12 slices of 4 mice, *** $p < 0.001$, Unpaired t test. **(D)** Schematic and representative images of RNA FISH co-staining of *Slc32a1*, *Meis2* and *Cacna2d1* mRNA in vlPAG area. Scale bars represent 50 μm. **(E)** Proportion of *Vgat*+ & *Meis2*+ neurons expressing *Cacna2d1* (NFD ctr: n = 550 cells from 12 slices of 4 mice, DIO: n =611 cells from 14 slices of 5 mice, **** $p < 0.0001$, Unpaired t test).

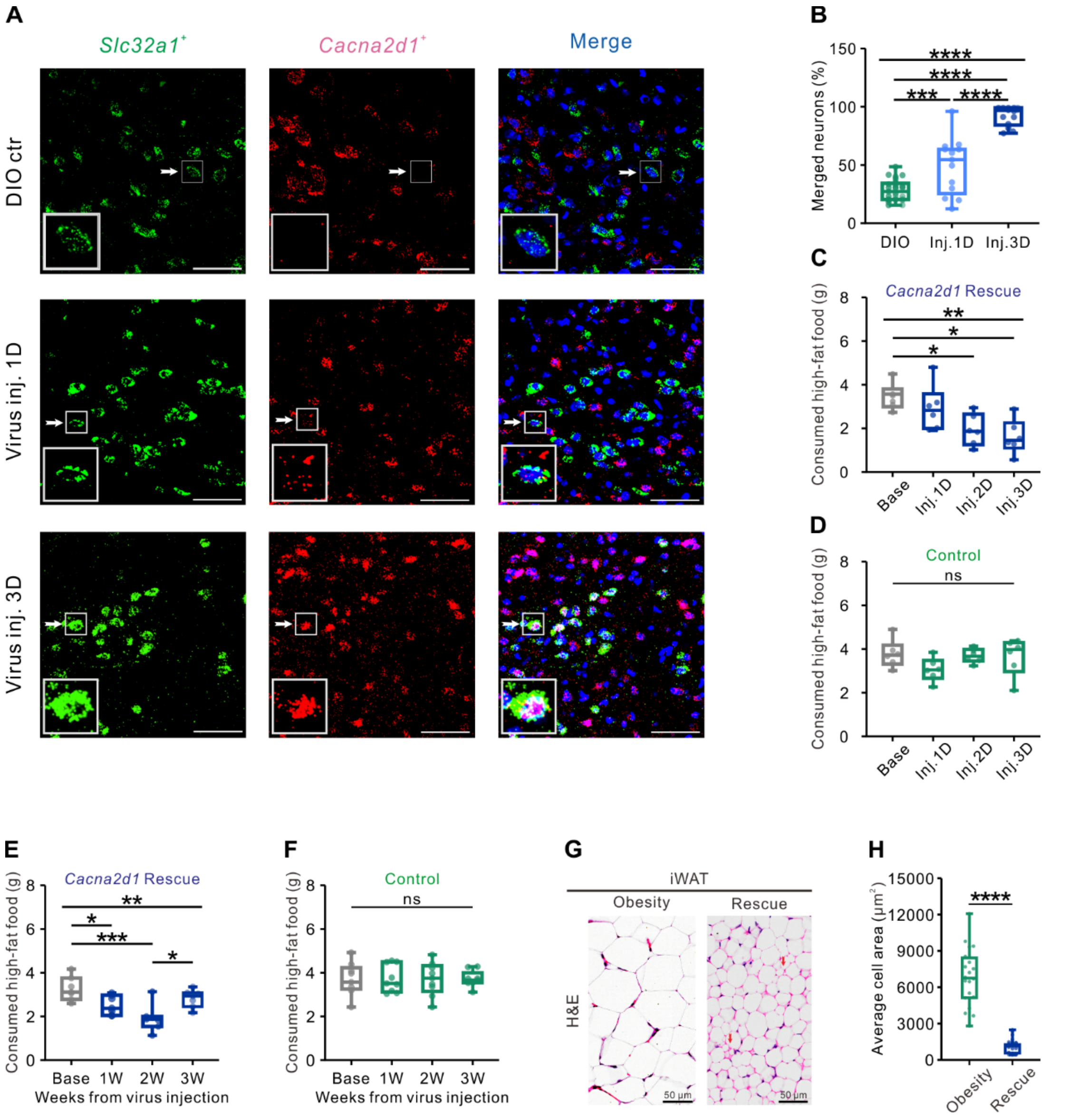

Fig. S13. Over-expression of CACNA2D1 inhibits food intake and increases browning of iWAT tissues.

(A) RNA FISH co-staining with *Slc32a1* and *Cacna2d1* in vlPAG area 1 day or 3 days after AAV-Cacna2d1 for injection. Scale bars represent 50 μm. **(B)** Proportion of *Vgat* positive neurons that expressing *Cacna2d1* (DIO ctr: n = 3369 cells from 27 slides of 4 mice; data were same from Fig. 4G, Inj. 1D: n = 653 cells from 13 slides of 4 mice, Inj. 3D: n = 646 cells from 14 slides of 4 mice, ***p < 0.001, ****p < 0.0001, one-way ANOVA with Tukey's multiple test). **(C-F)**

24 h food intakes during baseline and different time points after the injection of vehicle AAV-Cacna2d1 or vehicle AAV-eGFP (C&D: *Cacna2d1* rescue: n = 6 mice, HFD 7W control: n = 6 mice, *p < 0.05, **p < 0.01, one-way ANOVA with Tukey's multiple test; E&F: *Cacna2d1* rescue: n = 7 mice, HFD 7W control: n = 9 mice, *p < 0.05, **p < 0.01, *** p < 0.001, one-way ANOVA with Tukey's multiple test). **(G)** Representative H&E staining of iWAT from DIO mice after 3 weeks AAV-Cacna2d1 or AAV-eGFP injection into the vlPAG. Scale bars represent 50 μm. **(H)** Average adipocyte area in iWAT in DIO mice described in (G) (n = 20 slides from 4 mice for both groups; ****p < 0.0001, unpaired t test).

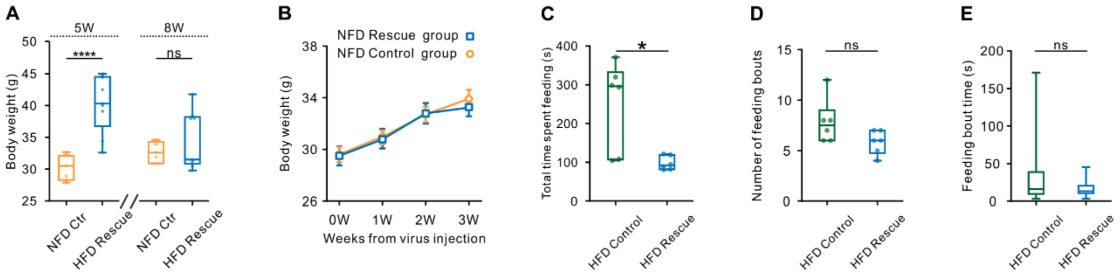

Fig. S14. Over-expression of CACNA2D1 reverses obesity in DIO mice, but does not reduce body weight in NFD mice.

(A) Body weights of CACNA2D1 over-expressed mice and age-matched eGFP-expressed mice fed with NFD were measured before and at the end of virus injection (NFD Ctr: n= 7 mice, HFD rescue: n= 9 mice; ****p < 0.0001, unpaired t test). **(B)** Body weight in mice fed with NFD after the injection of AAV-Cacna2d1 or AAV-eGFP (NFD ctr: n = 7 mice, NFD rescue: n = 6 mice; Data are represented as mean ± SEM; p = 0.7843, two-way repeated-measures ANOVA). **(C-E)** Total time the mice spent feeding (C), number and the length of feeding bouts (D and E respectively) during a 10 min test (HFD control Group: n = 6 mice, HFD rescue Group: n = 6 mice, C: *p < 0.05, D: p = 0.0802, E: p = 0.2101, unpaired t test or Mann-Whitney test).

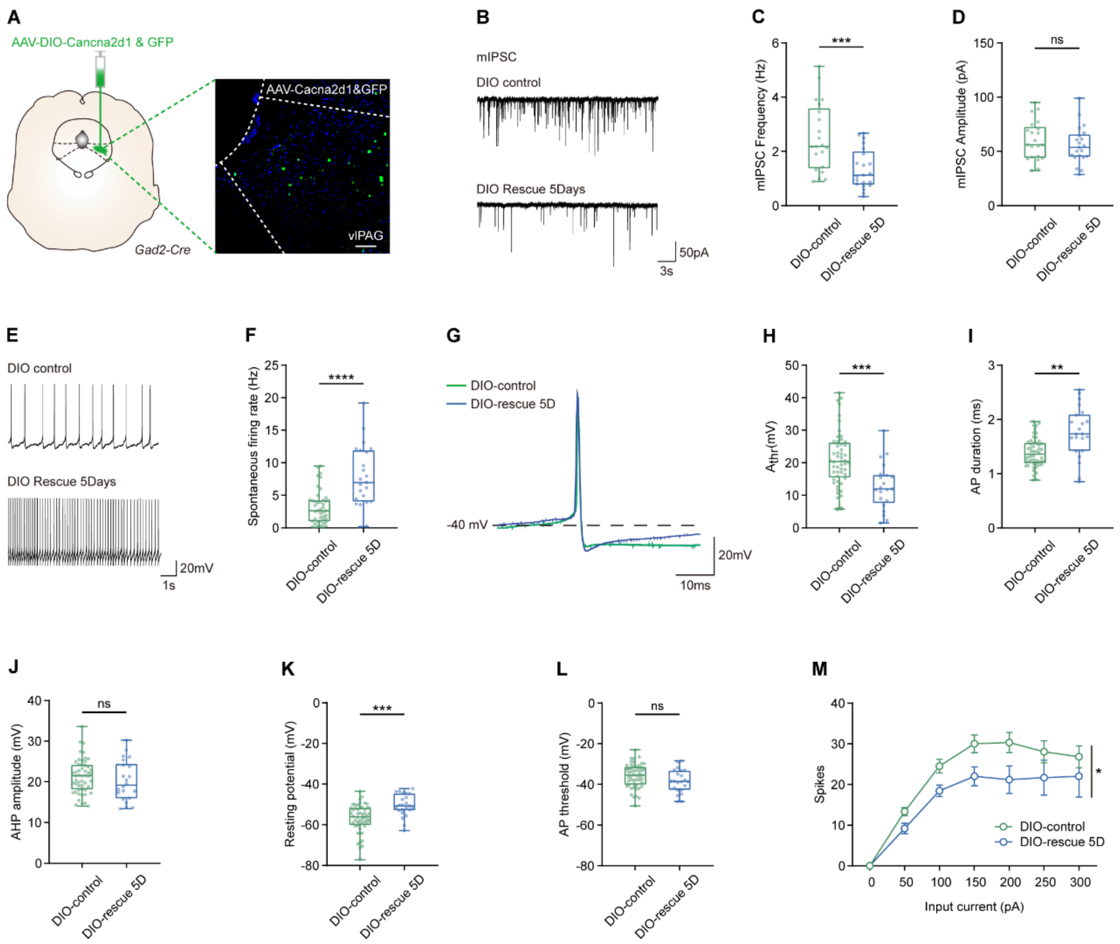

Fig.S15. Five days after CACNA2D1 over-expressing in DIO mice alters electrophysiological properties of vlPAG GABAergic neurons.

(A) Diagram of virus injection (left) and sample confocal images after 5 days Cacna2d1&GFP expressing in vlPAG GABAergic neurons (right). Scale bar represents 100 μm. **(B)** Representative traces of mIPSCs recorded in vlPAG GABAergic neurons from DIO control mice and DIO mice after 5 days over-expressing of CACNA2D1 (DIO-rescue 5D). **(C)** Average mIPSCs frequency from the two groups (DIO-control: n = 21 cells from 3 mice, DIO-rescue 5D: n = 21 cells from 4 mice, ***p < 0.001, unpaired t test). **(D)** Average mIPSCs amplitude from the two groups (p = 0.417, unpaired t test). **(E)** Representative

traces of spontaneous action potential (AP) firing from the two group. **(F)** Spontaneous AP firing rate of vlPAG GABAergic neurons from the two group (DIO-control: n = 43/52 cells from 9 mice, DIO-rescue 5D: n = 23/23 cells from 4 mice; ****p < 0.0001, Mann-Whitney test). **(G)** Representative traces of single AP. Dot lines represent -40mV. **(H-L)** Activation threshold (H, ***p < 0.001), action potential duration (I, **p < 0.01), AHP amplitude (J, p = 0.29), resting potential (K, ***p < 0.001) and AP threshold (L, p = 0.07) of vlPAG GABAergic neurons (DIO-control: n = 52 cells from 9 mice, DIO-rescue 5D: n = 23 cells from 4 mice, unpaired t test). **(M)** The number of evoked action potentials against injected currents (Data are represented as mean ± SEM ,* p < 0.05, two-way ANOVA followed by Holm-Sidak post hoc test).

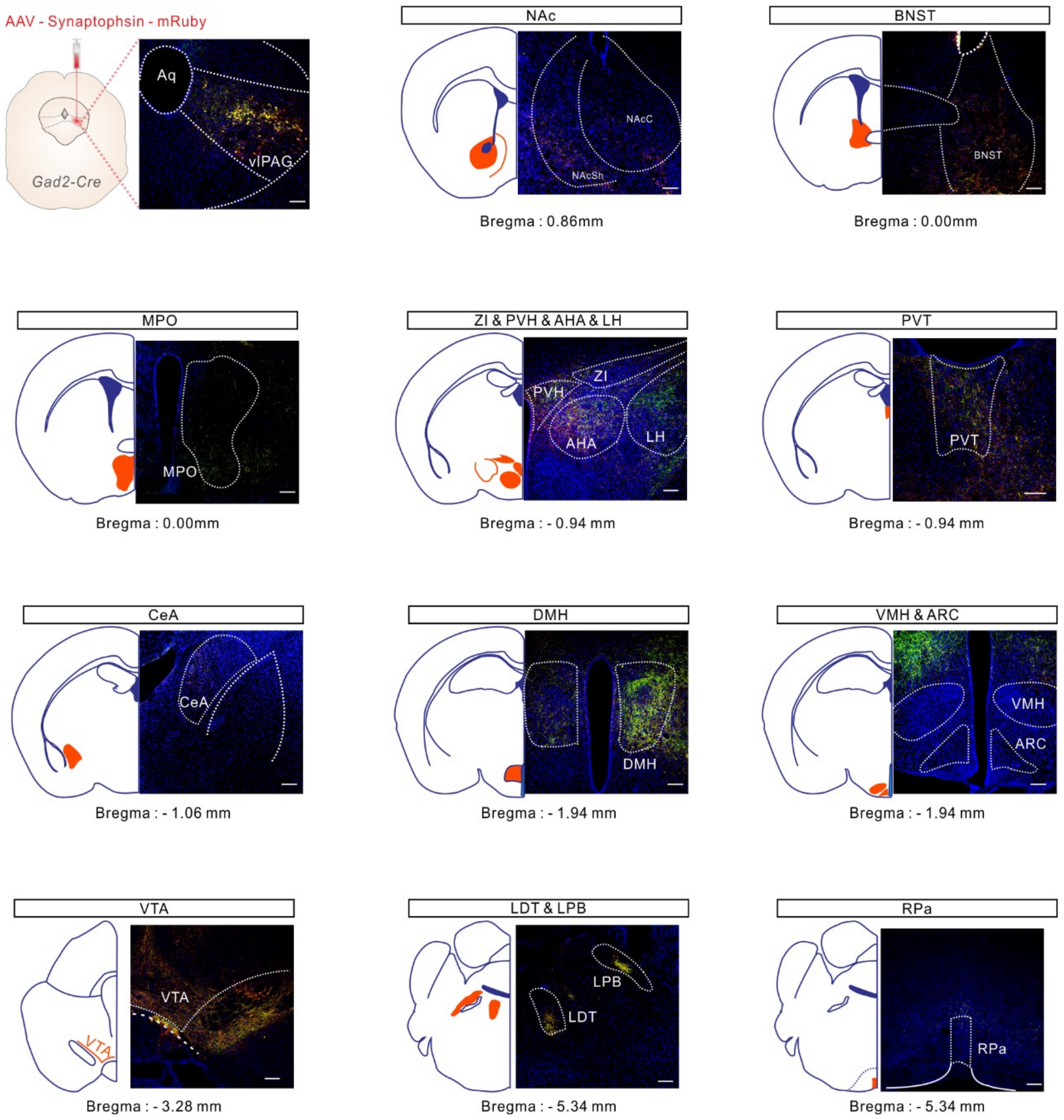

Fig. S16. vlPAG GABAergic neurons project to brain regions that implicated in feeding behavior or adipose tissue browning.

Coronal representations of anterograde monosynaptic labeling of vlPAG GABAergic axon terminals in feeding regulatory and adipose metabolism regulatory centers including the nucleus accumbens (NAc), the bed nucleus of the stria terminalis (BNST), the medial preoptic nucleus (MPO), the zona incerta (ZI), the paraventricular hypothalamic nucleus (PVH), the anterior hypothalamic

area (AHA), the lateral hypothalamic area (LH), the paraventricular thalamic nucleus (PVT), the central amygdaloid nucleus (CeA), the dorsomedial hypothalamic nucleus (DMH), the arcuate hypothalamic nucleus (ARC), the ventromedial hypothalamus (VMH), the ventral tegmental nucleus (VTA), the laterodorsal tegmental nucleus (LDT), the lateral parabrachial nucleus (LPB), and the raphe pallidus nucleus (RPa) (n=4 mice).

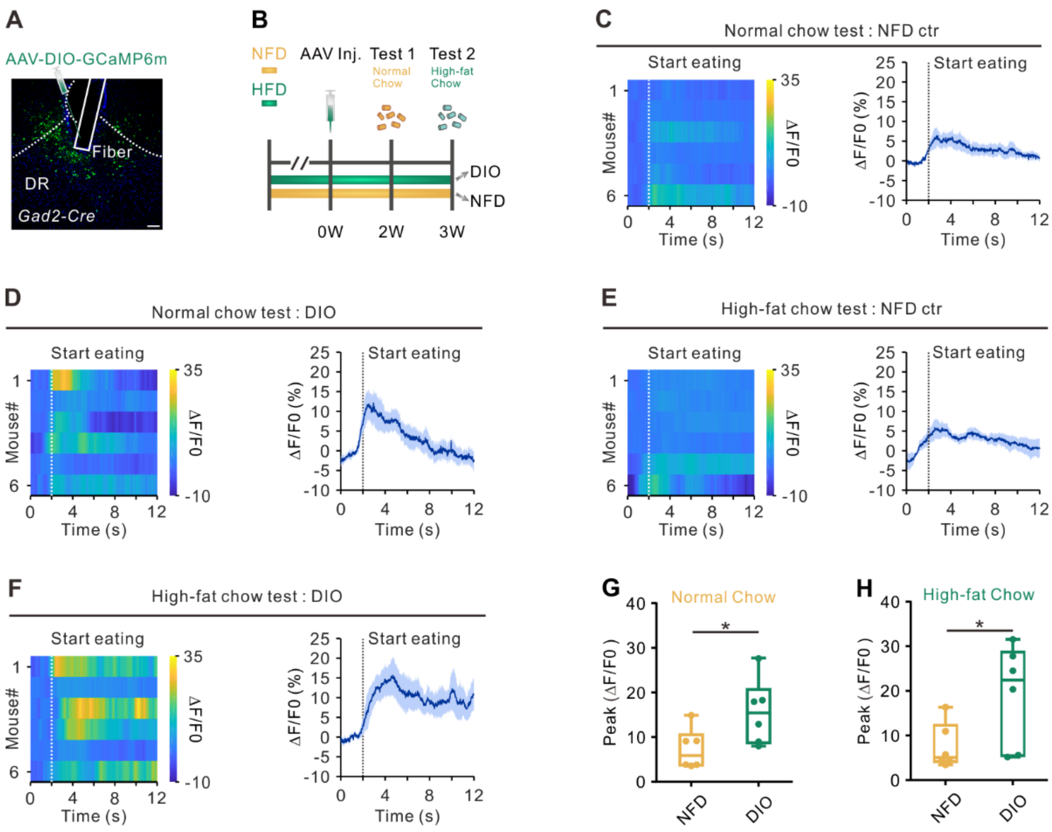

Fig. S17. Calcium imaging of DRN GABAergic cells during refeeding of normal chow or high-fat chow.

(A) Schematic for expressing GCaMP6m in $DRN^{GABAergic}$ neurons in *Gad2-Cre* mice. Scale bar represents 100 μm. **(B)** Experimental timeline and group schematic. **(C - D)** Heat maps showing the Ca^{2+} signals in DRN GABAergic neurons of NFD control mice (C) or DIO mice (D) in response to normal chow stimuli and corresponding mean GCaMP6m signal of all mice aligned to the initiation of feeding (n = 6 mice). **(E - F)** Heat maps showing the Ca^{2+} signals in DRN GABAergic neurons of NFD control mice (E) or DIO mice (F) in response to high-fat chow stimuli and corresponding mean GCaMP6m signal (n = 6 mice). **(G - H)** Amplitude of Ca^{2+} signaling in $DRN^{GABAergic}$ neurons in different groups (NFD ctr group: n = 6 mice, DIO group: n = 6 mice, $^*p < 0.05$, unpaired t test).

Cluster 1

Gene	p_val	avg_logFC
Malat1	7.67E-45	-0.4267928
Hsph1	2.20E-39	0.357978094
Stip1	1.50E-25	0.279331041
Cacna2d1	9.77E-19	-0.815887155
Nkain2	9.00E-14	-0.263342105
Dpf3	7.18E-13	0.255494907
Nckap5	2.79E-12	0.314802411
Slit3	8.83E-12	0.317628351
A230006K03Rik	2.83E-11	-0.314756307
Nell1	1.29E-10	0.296571724
Gm32647	2.97E-10	-0.499557681
Efna5	3.68E-10	0.2707775
Sox2ot	2.74E-08	-0.300949125
Adamtsl1	5.47E-08	0.315380571
Prr16	6.33E-08	0.288086548
mt-Rnr2	8.40E-08	-0.326171999
Hs3st4	1.01E-07	0.278709948
Zfpm2	1.25E-07	0.267994719
Galntl6	1.52E-07	-0.262874416
Trhde	3.06E-07	0.295157863
Fam19a1	1.18E-05	0.397633096
Arhgap26	1.27E-05	-0.251531115
Nfia	2.39E-05	0.268725238
Tox	5.41E-05	-0.336685695
mt-Rnr1	0.000187898	-0.290525894
Foxp2	0.000329721	-0.360495352
Pde10a	0.000855498	0.303272102
Gm20754	0.000945077	-0.251932432

Cluster 2

Gene	p_val	avg_logFC
Malat1	1.04E-29	-0.39198942
Hsph1	1.58E-17	0.327984294
Hspa4l	2.26E-15	0.365898453
Swt1	5.15E-15	0.277607879
Sik3	5.76E-13	0.266742192
Fxr1	5.50E-12	0.252688681
Pcsk1n	3.53E-10	-0.52218777
Efna5	1.53E-07	0.289277932
Pde10a	2.32E-07	0.456178041
Arhgap26	5.53E-07	-0.408420073
Alcam	1.18E-06	0.265469112
Thsd4	1.29E-06	0.300365853
Vwc2	1.42E-06	0.251187268
mt-Rnr2	4.83E-06	-0.251261865
Mir670hg	4.84E-06	0.279920598
Ndfip1	2.53E-05	-0.250265611
Cst3	3.67E-05	-0.381594166
Psap	0.000267854	-0.289577939
Stmn3	0.000721705	-0.311093952

Cluster 3

Gene	p_val	avg_logFC
Ahi1	6.63E-15	-0.315901731
Malat1	1.08E-10	-0.404999638
Swt1	2.31E-09	0.30451738
Hsph1	2.33E-09	0.321803938
Negr1	3.00E-09	0.274100254
Hspa4l	1.65E-08	0.397499819
Nxph1	2.43E-08	0.252050833
Grin3a	3.98E-07	0.291801461
Gm32442	6.16E-07	0.329358221
Kcnj3	9.05E-07	0.296069413
Fxr1	4.84E-06	0.253930317
Sema6a	8.74E-06	0.300498582
Prpf4b	1.80E-05	-0.27201228
Trps1	2.17E-05	0.319094789
mt-Rnr2	2.35E-05	-0.457953459
Pcdh7	6.28E-05	0.298784005
Atp6v0b	0.000107261	-0.31423785
Shisa9	0.000142852	0.251161967
Cntnap5b	0.000169259	0.315655024
A830018L16Rik	0.000187925	0.250741668
Chsy3	0.000237638	0.332131332
mt-Nd1	0.000370515	-0.474302943
Serpinb6a	0.000382274	-0.289825556
Cntnap5c	0.000682544	0.283436916
Khdrbs2	0.000772761	0.25769501
mt-Cytb	0.000974582	-0.431630557

Cluster 4

Gene	p_val	avg_logFC
Ahi1	1.09E-11	-0.356054921
Malat1	4.11E-11	-0.339827103
Lin7a	2.97E-08	0.412233162
mt-Rnr2	2.22E-07	-0.561670905
Rgs7	3.58E-06	0.254326546
Anks1	6.24E-06	0.261971822
Fxr1	1.30E-05	0.282025974
Kcnj3	2.32E-05	0.259219887
Tfcp2	2.87E-05	0.280352167
Mboat2	3.03E-05	0.255401902
Prpf4b	4.30E-05	-0.28375302
Aff3	7.85E-05	0.290652119
Magi3	0.000181034	0.267396147
Fat1	0.000293065	0.272877408
Top1	0.000543844	0.25710625
B230303A05Rik	0.000789123	-0.334808443
Megf9	0.000841755	0.256056458
Golgb1	0.000939407	-0.353045264
Mllt3	0.000961062	0.250485943
mt-Cytb	0.000973168	-0.439887389

Cluster 5

Gene	p_val	avg_logFC
Malat1	1.98E-07	-0.32432797
mt-Cytb	3.79E-06	-0.641017808
Lncpint	4.40E-06	0.341619265
Ahi1	1.74E-05	-0.31211709
Pdia3	5.44E-05	0.32102045
Chordc1	6.61E-05	0.261001028
Creb1	8.66E-05	0.28882407
Nptn	0.00010126	0.271410584
mt-Rnr2	0.000121509	-0.627395967
Lin28b	0.000126903	0.344290569
Mpdz	0.000134244	0.259334506
Sbf1	0.000190883	0.251408956
Chchd6	0.000193401	-0.341973925
Hspa4l	0.000217377	0.321284898
Stat5b	0.000220899	0.288205289
mt-Nd4	0.000338692	-0.505243577
Mboat2	0.000390606	0.301803743
Klhl2	0.000436303	0.251039824
Vav2	0.000448179	0.262194211
mt-Nd2	0.000582716	-0.488712305
Vegfb	0.000640273	-0.275547406
Cyp51	0.000651609	-0.338455825
Cpne5	0.000703412	0.250304283
4-Mar	0.000729962	0.255518258
mt-Nd1	0.000751846	-0.576352075
Dcun1d4	0.000904619	-0.312948865
Rbm39	0.000915318	-0.261979541

Cluster 6

Gene	p_val	avg_logFC
Ahi1	2.15E-09	-0.360497674
Malat1	6.64E-08	-0.450872468
Hsph1	2.92E-07	0.486014955
Gm37899	3.67E-06	0.320362045
Soga1	5.24E-06	0.254699586
Swt1	8.21E-06	0.369198375
Slc1a4	1.15E-05	0.270963005
Ptpro	1.39E-05	0.380600139
Lncpint	2.37E-05	0.269479868
Rbpj	2.78E-05	0.279484527
Epha7	2.93E-05	0.414977843
Castor2	3.44E-05	0.275317927
Atxn2l	4.93E-05	0.289970448
Ube2e3	7.49E-05	0.259858622
Cntnap5c	8.64E-05	0.394224282
Fmnl2	9.55E-05	0.400779757
Rfx3	0.000103469	0.307369163
Htr4	0.000107573	0.267070595
Dnaja1	0.000128554	0.310918934
Rbm39	0.000140054	-0.257774103
Lrch1	0.00014095	0.257472344
Tsc22d1	0.000155976	0.287399756
Fam189a1	0.000156107	0.287210317
Dlg4	0.000238453	0.314362418
Ppm1e	0.000325886	0.270954469
Sarnp	0.000340535	0.253983782
Pid1	0.000386636	0.29746285
Edil3	0.000402749	0.267063512
Arhgap24	0.000485595	0.31819904
Cdc123	0.000713889	0.255058706
Gpr137c	0.000818769	0.264028428
Dync2h1	0.000861411	-0.301094108

Cluster 7

Gene	p_val	avg_logFC
Parm1	1.69E-09	0.632973414
Eps15	2.58E-07	0.379729607
Hsph1	5.23E-07	0.505945628
Per2	6.32E-07	0.340290914
Spon1	1.53E-06	0.455063125
Rian	3.39E-06	0.333610379
Hspa4l	4.48E-06	0.52986901
Phtf2	4.53E-06	0.425508776
Rab3c	5.11E-06	0.526711401
Hnrnpll	5.57E-06	0.335900159
Ptprk	6.15E-06	0.637524641
Kirrel3	7.70E-06	-0.962791131
Plpp4	1.42E-05	0.531739884
Pbx4	1.63E-05	0.257924432
Matr3	1.72E-05	0.383875654
Tln1	1.83E-05	0.333342643
Psmd11	2.04E-05	0.276490153

Gene	p_val	avg_logFC
Apba2	2.09E-05	0.33008167
Mtf2	3.01E-05	0.414170742
Gclc	3.31E-05	0.266564097
Tsc22d4	3.31E-05	0.257789991
Gpatch8	3.44E-05	0.368188101
Abhd8	3.68E-05	0.2566138
Pkp4	3.91E-05	0.439857017
Ugp2	4.12E-05	0.271348385
St8sia4	4.20E-05	0.294871562
Il1rapl2	4.24E-05	-0.807347328
Fam208b	4.33E-05	0.352762038
Stag1	4.44E-05	0.372489156
Sgcd	4.51E-05	0.675160655
Qser1	4.58E-05	0.334270933
Rit2	5.29E-05	0.450674939
Slc1a2	5.65E-05	0.281322596
Ikzf4	5.74E-05	0.286015012

Cluster 7

Gene	p_val	avg_logFC
Slc35d1	6.40E-05	0.307350625
Itgb8	6.45E-05	0.384438986
Unc13b	6.71E-05	0.277178844
Atp2b1	6.72E-05	0.428229517
Clpx	6.94E-05	0.276277579
Gm42418	7.48E-05	-0.484252334
Arhgap26	7.51E-05	-0.623413172
Kcnb2	8.92E-05	-0.617339749
Nova1	9.17E-05	0.397706572
Vamp4	9.89E-05	0.283533601
Mpdz	0.000103497	0.433435365
Ndst4	0.000105112	-0.658537732
Cntnap5c	0.000106228	0.490096509
Chordc1	0.000110226	0.48609017
Nkain2	0.00011276	-0.490703096
Bex3	0.000113022	0.317349816
Edil3	0.000120215	0.628649025
Dscaml1	0.000126042	0.369297859
Taf1d	0.000150563	0.349362915
2610005L07Rik	0.000150774	0.375398669
Lrrc8c	0.000156649	0.293257061
Pomt1	0.000160827	0.280294394
Mfsd4a	0.000170989	0.304724602
Kcnj6	0.00017328	0.491629886
Bcas2	0.000173306	0.325477708
Srrm2	0.000195728	-0.388825536
B230217J21Rik	0.000204549	0.29500342
Pex13	0.000209066	0.276978209
D1Ertd622e	0.000210865	0.280409352
Lman2l	0.000211214	0.363911279
Nr6a1	0.000226921	0.475796392
Arhgef18	0.000229247	0.279967982
Aff2	0.000242283	0.36265832
Ptk2	0.000250687	0.372481433
Gid4	0.000250744	0.256829309
Mthfd2l	0.000259456	0.322168955
Papolg	0.000269466	0.255184588

Gene	p_val	avg_logFC
Pbx3	0.000296309	0.544422245
Zfp52	0.000306378	0.255912108
Maml3	0.000325812	0.387282019
9330159F1[illegible]	0.00033283	0.283824729
Ahi1	0.000368271	-0.294865027
Anxa6	0.000386521	0.267324921
Lin28b	0.000406843	0.347845404
Qtrt1	0.000458104	0.269779171
Supt20	0.000464065	0.286165509
Nr6a1os	0.000480668	0.276449478
Exosc3	0.000493443	0.252712369
Lhx1os	0.000495725	0.254820254
9330182L0[illegible]	0.000496933	0.335455519
N4bp2	0.000515431	0.271499433
Mboat7	0.000524684	0.320496488
Rhbdl3	0.000527866	0.332397879
Mfhas1	0.000550188	0.274060852
Stk32b	0.000573403	0.286362595
Arid5b	0.000652213	0.273324087
Cnot6	0.000663222	0.268464561
Fam19a2	0.000686756	-0.752256144
Gm4258	0.000686817	0.317593218
Gm32647	0.000690458	-0.824862903
Bbs4	0.000743462	0.34152753
Snapc3	0.00075519	0.334060902
Fcho2	0.000771408	0.359141772
1110019D1[illegible]	0.000774612	0.303058786
Dcaf1	0.000784348	0.273257349
Sec61a2	0.000811762	0.348696634
Ip6k1	0.000816169	0.286191201
Ranbp9	0.000828709	0.369482127
mt-Rnr2	0.000874551	-0.621643511
Dpysl5	0.000883108	0.380335833
Eif2s2	0.000904362	0.381191011
Sae1	0.000905126	0.277397189
Kcnd3	0.000908399	0.335275157
Cbl	0.000949371	0.256603602

Table. S1

HFD induces gene expression changes in the vlPAG.

A comprehensive list of genes that showing significant expression alterations in different vlPAG GABAergic cells clusters between WT and DIO.
